# Immune Profiling Identifies Inflammatory Signatures in Immune Checkpoint Inhibitor–Related Myocarditis

**DOI:** 10.1016/j.jaccao.2026.04.008

**Published:** 2026-06-16

**Authors:** Douglas Daoudlarian, Sarah Boughdad, Robin Bartolini, Sofiya Latifyan, Jacqueline Doms, Hasna Bouchaab, Karim Abdelhamid, Nabila Ferahta, Nuria Neisy Mederos Alfonso, Victor Joo, Antonia Stamatiou, Lucrezia Mencarelli, Nicolas Etienne, Athina Stravodimou, Khalil Zaman, Matthieu Perreau, Craig Fenwick, Keyvan Shabafrouz, Giuseppe Pantaleo, Solange Peters, Michel Obeid

**Affiliations:** a Centre Hospitalier Universitaire Vaudois (CHUV), University of Lausanne, Department of Medicine, Immunology and Allergy Service, Lausanne, Switzerland; b Nuclear Medicine Department, Groupe Hospitalier Pitié-Salpêtrière, Assistance Publique-Hôpitaux de Paris (AP-HP), Sorbonne Université, Paris, France; c Centre Hospitalier Universitaire Vaudois (CHUV), University of Lausanne, Department of Oncology, Medical Oncology Service, Lausanne, Switzerland

**Keywords:** biomarkers, immune checkpoint inhibitors, myocarditis, tocilizumab

## Abstract

**Background:**

Immune checkpoint inhibitor–associated myocarditis (ICI-My) is rare but potentially life-threatening. Biomarkers that distinguish myocarditis-related inflammation from background immune activation induced by ICIs remain needed.

**Objectives:**

This study sought to define the circulating inflammatory and cellular immune landscape of ICI-My, relate these findings to clinical severity, and explore the feasibility of interleukin-6 receptor (IL-6R) blockade in selected steroid-refractory cases.

**Methods:**

In this single-center retrospective cohort (January 2018 to June 2024), we performed biomarker profiling including multiplex cytokine profiling in 33 patients with ICI-My (7 severe and 26 nonsevere, 28 cytokines profiles) and 68 ICI-treated patients without myocarditis or other immune-related adverse events. Mass cytometry analyses compared 16 patients with ICI-My with 72 ICI-treated patients without myocarditis or other immune-related adverse events. We also describe eight steroid-refractory patients treated with tocilizumab on a compassionate-use basis.

**Results:**

Compared with ICI-treated controls, ICI-My was associated with higher circulating IL-6, CCL3, CCL4, CCL5, CXCL9, CXCL10, CXCL13, and VEGF-A. Mass cytometry analyses showed qualitative immune-cell differences, including higher proportions of immature neutrophils and activated HLA-DR^+^CD38^+^ CD4^+^ and CD8^+^ T cells, lower CXCR5^+^ memory B- and T-cell populations, contraction of switched and unswitched memory B-cell compartments, and lower CXCR3 expression across memory T-cell subsets. No clear systemic complement activation signal was observed. High-sensitivity troponin T, N-terminal pro–B-type natriuretic peptide, aspartate aminotransferase, and alanine aminotransferase discriminated severe from nonsevere myocarditis more consistently than individual cytokines. In eight steroid-refractory cases, tocilizumab administration was feasible, but this uncontrolled series cannot establish efficacy.

**Conclusions:**

Peripheral immune profiling identifies an IL-6–centered and chemokine-centered inflammatory signature in ICI-My beyond background ICI exposure. Conventional cardiac biomarkers remain more informative than single cytokines for severity assessment in this cohort. IL-6R blockade appears biologically plausible and clinically feasible in selected steroid-refractory cases and warrants prospective evaluation.

## Introduction

Immune checkpoint inhibitor–associated myocarditis (ICI-My) is an uncommon but potentially life-threatening toxicity of cancer immunotherapy, with an estimated incidence near 1% and high early mortality in historical series.^1^, ^2^, ^3^ Despite growing awareness, systematic troponin surveillance in some centers, and improving diagnostic frameworks, ICI-My remains challenging to diagnose, risk stratify, and manage.^4^^,^^5^ The pathophysiology of ICI-My likely reflects loss of peripheral tolerance and a coordinated inflammatory response involving T cells, myeloid cells, B cells, and soluble mediators. Cardiac tissue studies have highlighted lymphocytic and macrophage infiltrates, but the circulating immune signature of ICI-My remains incompletely defined, particularly in comparison with patients receiving ICI therapy without myocarditis.^3^^,^^6^ This distinction is biologically important because ICI exposure itself reshapes the peripheral immune landscape.

Recent profiling studies across immune-related toxicities have implicated interleukin-6 (IL-6) and interferon-γ–inducible chemokines, such as CXCL9 and CXCL10, in severe inflammatory syndromes.^7^, ^8^, ^9^, ^10^ However, few studies have combined broad cytokine profiling with deep peripheral immune phenotyping in ICI-My and compared these findings with those in ICI-treated control patients without myocarditis.^10^, ^11^, ^12^

In this retrospective single-center study, we characterized circulating cytokines, immune-cell subsets, clinical severity, and a small real-world series of IL-6R blockade in steroid-refractory cases (Central Illustration). Our analyses were designed to be hypothesis-generating and to inform prospective studies.

**Central Illustration:**
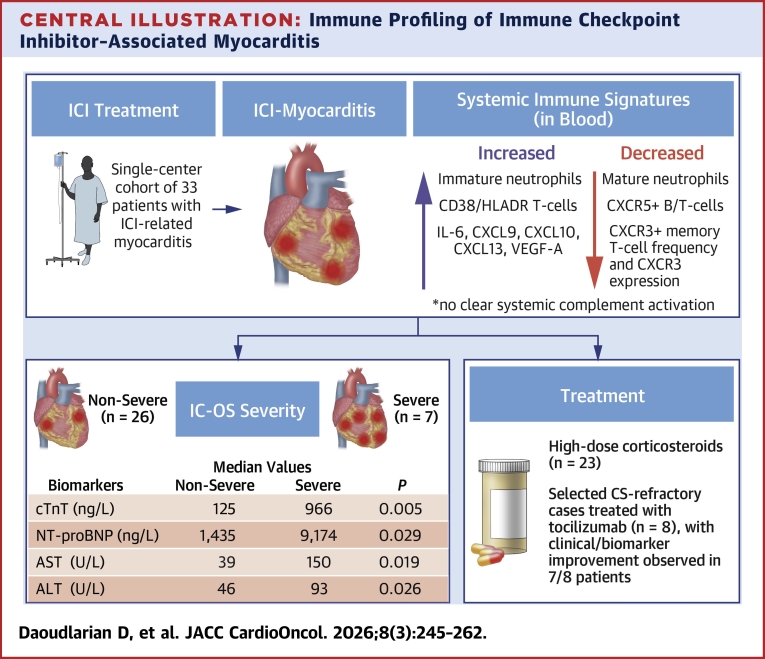
Immune Profiling of Immune Checkpoint Inhibitor–Associated Myocarditis In this single-center cohort of 33 patients with immune checkpoint inhibitor (ICI)–associated myocarditis, comparison with biologically appropriate ICI-treated controls without myocarditis identified a systemic IL-6–centered and chemokine-centered inflammatory signature. This signature was characterized by higher proportions of immature neutrophils and activated T cells, lower CXCR5^+^ memory B- and T-cell populations, lower CXCR3 expression across memory T-cell subsets, and no clear systemic complement activation signal. Conventional cardiac biomarkers and electrical instability better discriminated severe from nonsevere disease than individual cytokines. In selected steroid-refractory cases treated with tocilizumab, clinical and biomarker trajectories were generally favorable, but prospective validation is required.

## Methods

### Patient consent, ethical approval, and sample collection

The study was approved by the Cantonal Ethics Committee of Vaud (CER-VD) under the Immuno-TOX protocol (CER-VD 2022-01030) and conducted in accordance with the Swiss Federal Act on Research involving Human Beings. Biological samples were collected during routine clinical care. All participants provided consent for the research use of coded clinical data and biospecimens through the institutional general consent process, or where applicable, inclusion was authorized under Article 34, as specified in the approved protocol. The ICI-treated control cohort was identified through the Lausanne Center for Immuno-Oncology Toxicities registry (CHUV_2022_016_RM), which was approved by the same ethics authority; written informed consent was obtained from all included participants. Cytokine and CyTOF data from ICI-treated control patients were obtained under the Biomark protocol (CER-VD 2019-02422), for which all participants provided specific written informed consent for research use.

### Study design and patient cohort

This retrospective study was conducted at Centre Hospitalier Universitaire Vaudois (CHUV) across the Medical Oncology, Immunology and Allergy, and Cardiology services. We included all patients diagnosed with ICI-My between January 2018 and June 2024 based on the prespecified diagnostic criteria discussed in the following section. The myocarditis cohort comprised 33 patients. For cytokine analyses, patients with ICI-My were compared with 68 ICI-treated patients without myocarditis or other immune-related adverse events (irAEs). For mass cytometry analyses, 16 patients with ICI-My were compared with 72 ICI-treated patients without myocarditis or other irAEs. Patients with alternative causes of myocarditis, including infectious myocarditis, were excluded.

### Diagnostic criteria for ICI-My

ICI-My was defined according to the 2022 European Society of Cardiology cardio-oncology guidance.^13^ All included cases fulfilled these criteria. Diagnostic certainty (definite, probable, or possible) was additionally adjudicated using the Bonaca framework and is reported in detailed patient-level tables, but Bonaca classification was not used as an inclusion criterion.^14^ Clinical severity was classified according to the 2022 International Cardio-Oncology Society (IC-OS) consensus definition.^15^ Severe ICI-My was defined as myocarditis with any of the following condition: hemodynamic instability, heart failure requiring ventilatory support, complete or high-grade heart block, or significant ventricular arrhythmias; all other cases were classified as nonsevere.^5^^,^^15^ The standardized diagnostic workup included high-sensitivity (hs) troponin T and/or I, creatine kinase (CK), CK-myocardial band (CK-MB), N-terminal pro–B-type natriuretic peptide (NT-proBNP), electrocardiography, transthoracic echocardiography, and cardiac magnetic resonance (CMR) imaging when feasible. Coronary angiography and endomyocardial biopsy were performed selectively after multidisciplinary review. Patients were also screened for overlapping neuromuscular irAEs, including myositis and myasthenia gravis. Our center's routine high-frequency troponin surveillance in ICI-treated patients facilitates detection of early-stage myocarditis^15^ and likely contributed to the identification of smoldering or nonsevere cases in this cohort. This approach may have favored earlier diagnosis and intervention;^16^ however, whether it reduces long-term major adverse cardiovascular events requires confirmation in dedicated prospective studies.^4^

### Management of immunomodulatory treatment and corticosteroid-refractory or less-responsive severe ICI-My

High-dose corticosteroids were used as first-line therapy in accordance with current expert guidance.^17^ We considered severe ICI-My less responsive to corticosteroids when, despite at least 48 hours of high-dose corticosteroids (>1 mg/kg/day prednisone equivalent), with or without other second-line agents, patients had persistent or worsening hemodynamic compromise, a 25% or greater rise in cardiac biomarkers, malignant or hemodynamically significant arrhythmias, or progressive ventricular dysfunction on imaging. In selected cases, we escalated to tocilizumab (TCZ) to target the IL-6 pathway while avoiding broader immunosuppressive strategies when clinically appropriate. This institutional approach is supported by the European Society for Medical Oncology guidelines and accumulating clinical evidence.^10^, ^11^, ^12^^,^^18^ At our institution, TCZ was generally administered intravenously at 8 mg/kg every 2 weeks until biochemical and clinical stabilization or at the treating team's discretion.

### Statistical analysis

Continuous variables are presented as median (Q1–Q3), unless otherwise specified, and categorical variables as count (percentage). Comparisons between groups were performed using the Mann-Whitney *U* test or Fisher's exact test, as appropriate. Correlations were assessed using Spearman rank correlation coefficient. Cytokines at the lower limit of detection were handled as specified in Supplemental Methods. For cytokine analyses, *P* values were adjusted for multiple pairwise comparisons using the Benjamini–Hochberg false discovery rate procedure, with q values reported in Supplemental Tables. Immune-cell subset comparisons were considered exploratory and were not adjusted for multiple testing. Statistical analyses were performed using GraphPad Prism version 10.1.2 (GraphPad Software), MATLAB R2024a (MathWorks), and FlowJo version 10.9.0 (BD Life Sciences). All statistical tests were two-sided, and *P* values are reported to three decimal places, with values below 0.001 reported as *P* < 0.001.

### Supplemental methods

Protocols for serum biomarker measurement and cellular phenotyping are provided in Supplemental Methods. The antibody list and gating strategy for mass cytometry are detailed in Supplemental Figure 1 and Supplemental Methods.

### Data availability

Descriptive statistics for cytokine data have been provided for the main figures. Corresponding FCS files/source data and deidentified patient-level cytokine data are available from the corresponding author upon request, in accordance with applicable ethical and institutional requirements.

## Results

### Study population

A total of 33 patients with ICI-My were included (Supplemental Figure 2). Patient-level clinical characteristics are provided in Table 1. Using IC-OS severity criteria, 7 patients (21%) were classified as severe, and 26 (79%) as nonsevere. A clinical syndrome compatible with myocarditis was present in all patients (33 of 33, 100%) (Table 2). Symptom burden varied, with one symptom in 12 patients (36%), two symptoms in 11 (33%), and more than two symptoms in 10 (30%) (Tables 2 and 3). Evidence of decreased systolic cardiac function was observed in 15 patients (45%), and ventricular arrhythmia and/or conduction system disease was observed in 10 (30%) (Table 2).

**Table 1:** Detailed Patient Characteristics

Patient ID (n = 33)	Age (years, range)	Sex	Cancer type	Cancer stage (TNM)	ICI treatment	Time to symptom onset from ICI start (weeks)	Presenting symptoms	IC-OS myocarditis grading	Immune-related adverse events	Prior anticancer therapies	ECG key finding	Ventricular arrhythmia (VT/VF)	ECG phenotype	Transthoracic echocardiography (TTE)	Coronary angiography	Cardiac MRI status	Cardiac MRI keywords	Bonaca classification	Max steroid dose	Tocilizumab (8 mg/kg IV)	Mycophenolate mofetil (1g BID)	Anti-TNF (infliximab, 5 mg/kg IV)	Other immunosuppressants
Tocilizumab Patient 1	70-75	F	Pulmonary adenocarcinoma	IVB	Pembrolizumab	5	Chest pain	Nonsevere	Cholangiohepatitis	Chemotherapy	Bundle/fascicular block	No	Conduction system disease	Normal TTE	Not done	Not feasible	Not feasible	Possible	1 mg/kg	Yes (1 dose)	None	None	None
Tocilizumab Patient 2	50-55	M	Small-cell neuroendocrine lung carcinoma	IIIB	Ipilimumab-nivolumab	4	Pericardial tamponade, surgically drained, with symptoms	Severe	Ocular myositis	Radiochemotherapy/lurbinectedin	Complete AV block	No	Conduction system disease	LV dysfunction + pericardial effusion	Negative	Not performed	Not performed	Probable	Steroid bolus 500 mg/d (3 days) then 2 mg/kg	Yes (1 dose)	None	None	None
Tocilizumab Patient 3	65-70	M	Melanoma	IIIB	Ipilimumab-nivolumab	11	Fatigue, chest pain	Nonsevere	Colitis/duodenitis/gastritis/multiple mononeuropathy	Pembrolizumab	Normal ECG	No	Neither	Normal TTE	Negative	Positive	Nonischemic injury	Definitive	1 mg/kg	Yes (1 dose)	None	None	None
Tocilizumab Patient 4	35-40	F	Breast cancer	IIIC	Pembrolizumab	12	Fatigue, chest pain, eyelid edema	Nonsevere	Bilateral uveitis/nephritis/arthritis/pancytopenia/hepatitis/DIC	Chemo-immunotherapy	Normal ECG	No	Neither	Mild LV dysfunction	Not done	Not performed	Not performed	Probable	2 mg/kg	Yes (2 doses)	None	None	None
Tocilizumab Patient 5	45-50	M	Poorly differentiated renal carcinoma	IV	Ipilimumab-nivolumab	4	Fatigue	Nonsevere	Colitis	Radiation treatment	Normal ECG	No	Neither	Normal TTE	Not done	Not performed	Not performed	Possible	0.5 mg/kg	Yes (3 doses)	None	Yes (1 dose)	None
Tocilizumab Patient 6	85-90	M	Melanoma	IV	Ipilimumab-nivolumab	1	Chest pain with diaphoresis and hypotension, lower limbs odema	Severe	Colitis G3	Pembrolizumab	Right bundle branch block	No	Conduction system disease	LV dysfunction	Negative	Positive	Edema; nonischemic injury	Definitive	Steroid bolus 500 mg/d (3 days) then 1 mg/kg	Yes (3 doses)	None	None	None
Tocilizumab Patient 7	55-60	M	Melanoma	IV	Ipilimumab-nivolumab	18	Abdominal pain, fatigue	Nonsevere	Colitis G3/pancreatitis	Ipilimumab-nivolumab/encorafenib/binimetinib/radiation	Normal ECG	No	Neither	Normal TTE	Not done	Suggestive	Suspicious myocarditis pattern	Possible	Steroid bolus 500 mg/d (3 days) then 1 mg/kg	Yes (1 dose)	None	None	None
Tocilizumab Patient 8	65-70	M	Prostate adenocarcinoma	III	Ipilimumab-nivolumab	7	Cardiac arrest	Severe	Auto-immune neutropenia	Chemotherapy/radiation treatment/enzalutamide	High-grade AV block	No	Conduction system disease	LV dysfunction	Negative	Not performed	Not performed	Probable	Steroid bolus 500 mg/d (3 days) then 1 mg/kg	Yes (3 doses)	Yes (29 days)	None	None
Patient 9	65-70	F	Pulmonary adenocarcinoma	IIIB	Pembrolizumab	35	Fatigue, dyspnea	Nonsevere	Pneumopathy G3	Chemotherapy-nivolumab/chemotherapy-pembrolizumab/radiation	Sinus tachycardia	No	Neither	Normal TTE	Not done	Not performed	Not performed	Possible	1 mg/kg	None	None	None	Intravenous immunoglobulin
Patient 10	80-85	M	Melanoma	IV	Nivolumab	10	Fatigue, muscle soreness	Nonsevere	Myositis/diplopia	None	Normal ECG	No	Neither	Normal TTE	Not done	Negative	No myocarditis pattern	Possible	1 mg/kg	None	None	None	None
Patient 11	75-80	M	Hepatocellular carcinoma	IV	Pembrolizumab-quavonlimab	4	Fatigue, dyspnea	Nonsevere	Colitis/pancreatitis/maculopapular exanthema	Lenvatinib/transarterial radio-embolization	Sinus bradycardia	No	Neither	Normal TTE	Not done	Negative	No myocarditis pattern	Possible	Steroid bolus 500 mg/d (3 days) then 1 mg/kg	None	None	Yes (1 dose)	None
Patient 12	50-55	F	Cutaneous peripheral T lymphoma without specificity NOS	IVB	Pembrolizimab	6	Fatigue, muscle soreness	Severe	None	Chemotherapy/ruxolitinib/romidepsine/rituximab/infliximab	Complete AV block, pacing	No	Conduction system disease	LV dysfunction	Negative	Positive	Edema; nonischemic injury	Definitive	1 mg/kg	None	None	None	None
Patient 13	65-70	M	Pulmonary adenocarcinoma	IIIA	Durvalumab	3	Dyspnea with cardiogenic shock and cardiac arrest	Severe	Pneumopathy	Radiochemotherapy	Ventricular fibrillation	Yes	Ventricular arrhythmia	Severe biventricular dysfunction	Not done	Not performed	Not performed	Probable	2 mg/kg	None	None	Yes (3 doses)	None
Patient 14	75-80	M	Hepatocellular carcinoma	IV	Atezolizumab	15	Myositis-myocarditis overlap syndrome (ptosis + dysphagia), fatigue	Nonsevere	Myopathy/ptosis	None	Minor ST–T changes	No	Neither	Normal TTE	Not done	Negative	Negative	Possible	1 mg/kg	None	None	None	Pyridostigmine and pyridostigmin
Patient 15	45-50	M	Melanoma	IV	Pembrolizimab	25	Lower-limb edema, dyspnea	Severe	Thyroiditis	Ipilimumab-nivolumab/encorafenib-binimetinib/lenvatinib-pembrolizumab	Ventricular tachycardia	Yes	Ventricular arrhythmia	LV dysfunction	Not done	Suggestive	Suspicious myocarditis pattern	Definitive	0.5 mg/kg	None	None	None	Colchicine
Patient 16	70-75	M	Unclassifiable melanoma	IIIC	Pembrolizumab	5	Dyspnea, abdominal pain	Nonsevere	None	None	Normal ECG	No	Neither	Missing data	Not done	Not performed	Not performed	Possible	0.5 mg/kg	None	None	None	None
Patient 17	60-65	M	Melanoma	IIIC	Ipilimumab-nivolumab	12	Syncope, dyspnea, fatigue	Nonsevere	Immune-induced corticotropic and thyrotropic insufficiency	Nivolumab/radiation treatment	Normal ECG	No	Neither	Severe LV dysfunction	Not done	Not performed	Not performed	Possible	1 mg/kg	None	None	None	None
Patient 18	60-65	M	Anal canal squamous cell carcinoma	IV	Pembrolizumab	4	Muscle soreness, dyspnea, fatigue	Nonsevere	Myositis G3 Hepatitis G2	Radiochemotherapy	T-wave inversions	No	Neither	Normal TTE	Not done	Negative	No myocarditis pattern	Possible	Steroid bolus 500 mg/d (3 days) then 2 mg/kg	None	None	None	None
Patient 19	35-40	F	Lung adenocarcinoma	IVB	Atezolizumab	2	Chest pain, dyspnea, fatigue	Nonsevere	Choroidal venous vasculitis G3/VKH-like uveitic disease	Chemotherapy/alectinib/lorlatinib/Radiation treatment	Normal ECG	No	Neither	Normal TTE	Not done	Other/unclear	As reported	Possible	1 mg/kg	None	None	None	None
Patient 20	30-35	F	Squamous cell carcinoma of the oral cavity	III	Pembrolizumab	7	Chest pain	Nonsevere	Thyroiditis	pembrolizumab	ST–T changes	No	Neither	Normal TTE	Missing data	Negative	No myocarditis pattern	Possible	1 mg/kg	None	None	None	None
Patient 21	60-65	M	Melanoma	IIIC	Pembrolizumab	5	Fatigue, neck pain	Nonsevere	Hepatitis G3/myopathy G3	None	First-degree AV block ± fascicular block	No	Conduction system disease	Normal TTE	Not done	Negative	No myocarditis pattern	Possible	Steroid bolus 500 mg/d (3 days) then 1 mg/kg	None	Yes (59 days)	Yes (2 doses)	None
Patient 22	80-85	F	Pulmonary adenocarcinoma	IIIB	Durvulumab	50	Dyspnea, tachypnea, desaturation, lower limbs edema	Nonsevere	None	Chemotherapy/radiation treatment	Normal ECG	No	Neither	LV dysfunction	Not done	Negative	No myocarditis pattern	Probable	1 mg/kg	None	None	None	None
Patient 23	70-75	M	Pulmonary adenocarcinoma	IVA	Ipilimumab-nivolumab	17	Dyspnea, fatigue, feet edema	Nonsevere	Arthritis G2/myopathy G2	Chemotherapy	First-degree AV block	No	Conduction system disease	LV dysfunction	Not done	Negative	No myocarditis pattern	Probable	0.5 mg/kg	None	None	None	None
Patient 24	70-75	M	Pulmonary adenocarcinoma	IV	Pembrolizumab	12	Dyspnea and chest pain and associated pericarditis	Nonsevere	Pericarditis	Chemo-immunotherapy	ST–T changes	No	Neither	Severely dilated LV with dysfunction	Not done	Not feasible	Not feasible	Probable	N/A	None	None	None	None
Patient 25	45-50	M	Renal clear cells carcinoma	IV	Pembrolizumab	6	Fatigue, chest pain	Nonsevere	Myositis G3/hepatitis G1/thyroiditis	None	Normal ECG	No	Neither	Normal TTE	Not done	Positive	Nonischemic injury	Definitive	N/A	None	None	None	None
Patient 26	70-75	M	Tonsil squamous cell carcinoma	III	Pembrolizumab	14	Palpitations	Nonsevere	Cardiovascular event	Radiation treatment	Normal ECG	No	Neither	Missing data	Not done	Not performed	Not performed	Possible	N/A	None	None	None	None
Patient 27	60-65	M	Pulmonary adenocarcinoma	IVA	Nivolumab	32	Dyspnea, chest tightness, lower-limb edema	Nonsevere	Colitis	Chemotherapy-pembrolizumab/sitravatinib and nivolumab/radiation treatment	Normal ECG	No	Neither	LV dysfunction	Not done	Suggestive	Suspicious myocarditis pattern	Probable	N/A	None	None	None	None
Patient 28	80-85	M	Malignant pleural epithelioid mesothelioma	II	Nivolumab	25	Associated pericarditis	Nonsevere	Pneumonitis G3	Ipilimumab-nivolumab/radiation treatment	T-wave inversions	No	Neither	Normal TTE	Not done	Negative	No myocarditis pattern	Possible	N/A	None	None	None	None
Patient 29	75-80	F	Merkel carcinoma	IIA	Pembrolizumab	17	Muscular weakness chest/shoulder/lower-jaw pain, dyspnea, cardiac arrest	Nonsevere	SIADH/cardovascular event	Radiation treatment	Normal ECG	No	Neither	Normal TTE	Not done	Suggestive	Suspicious myocarditis pattern	Probable	N/A	None	None	None	None
Patient 30	55-60	M	Pulmonary squamous cell carcinoma	IV	Pembrolizumab	29	Associated pericarditis, chest and arm pain	Severe	Obstructive shock state	None	Sinus tachycardia	No	Neither	LV + RV dysfunction	Negative	Suggestive	Suspicious myocarditis pattern	Probable	N/A	None	None	None	None
Patient 31	65-70	M	Large-cell lung neuroendocrine carcinoma	IIB	Atezolizumab	8	Asymptomatic	Nonsevere	Colitis G3	Chemotherapy	First-degree AV block ± bundle branch block	No	Conduction system disease	LV dysfunction	Not done	Negative	No myocarditis pattern	Possible	N/A	None	None	None	None
Patient 32	65-70	M	Cutaneous squamous cell carcinoma	III	Cemiplimab	7	Fatigue, syncope	Nonsevere	None	None	ST–T changes	No	Neither	LV dysfunction	Not done	Not feasible	Not feasible	Possible	N/A	None	None	None	None
Patient 33	65-70	M	Lung adenocarcinoma and squamous cell carcinoma of the retropharynx	IV and III	Pembrolizumab	68	Asymptomatic	Nonsevere	Colitis/hyponatremia on SIADH1	Chemotherapy/radiation treatment	Supraventricular ectopy	No	Neither	Missing data	Not done	Not performed	Not performed	Possible	N/A	None	None	None	None

SIADH = Syndrome of Inappropriate Antidiuretic Hormone Secretion; LV = left ventricle; AV = atrioventricular; RV = right ventricle; ECG = electrocardiography; ICI = immune checkpoint inhibitor; IC-OS = International Cardio-Oncology Society; MRI = magnetic resonance imaging; VF = ventricular fibrillation; VKH = VKH = Vogt-Koyanagi-Harada; VT = ventricular tachycardia..

**Table 2:** Diagnostic and Severity Features of the ICI-My Cohort

	Nonsevere (n = 26)	% within cohort	Severe (n = 7)	% within cohort	*P*
Diagnostic features					
Histopathological evidence of myocarditis			0/1 (n = 1)		
Clinical-major^a^	2	8%	2	29%	0.190
Clinical-minor					
Clinical syndrome^b^	26	100%	7	100%	>0.99
1 clinical symptom	10	38%	2	29%	>0.99
2 clinical symptoms	8	31%	3	43%	0.68
>2 clinical symptoms	8	31%	2	29%	>0.99
Decline in cardiac (systolic) function	8	31%	7	100%	0.002 (∗∗)
Any ventricular arrhythmia and/or new conduction system disease	4	15%	6	86%	0.005 (∗∗)
Suggestive CMR	5	19%	2	29%	0.62
					Total
CMR: Clinical-Minor total	24		5		29 (88%)
CMR not performed	11	42%	3	43%	14 (42%)
CMR suggestive	5	19%	2	29%	7 (21%)
CMR negative	8	31%	0	0%	8 (24%)
Clinical determinants of myocarditis severity	0/26		7/7		<0.001 (∗∗∗)
Heart failure requiring ventilation (noninvasive or invasive)	0	0%	1	14%	
Hemodynamic instability	0	0%	3	43%	
Complete or high-grade heart block	0	0%	3	43%	
Significant ventricular arrhythmia	0	0%	2	29%	

a Presence of both nonischemic myocardial injury and myocardial edema on CMR, according to the modified Lake Louise criteria.

b Clinical syndrome includes any one of the following: fatigue, muscle weakness, myalgias, chest pain, diplopia, ptosis, shortness of breath, orthopnea, lower extremity edema, palpitations, lightheadedness/dizziness, syncope, cardiogenic shock. ∗*P* < 0.05, ∗∗*P* < 0.01, and ∗∗∗*P* < 0.001.

**Table 3:** Clinical Characteristics of ICI-My

	Nonsevere (n = 26)	% within cohort	Severe (n = 7)	% within cohort	*P*
Clinical presentation					
Chest pain	9	35%	3	43%	0.69
Pain	10	38%	3	43%	>0.99
Palpitations	3	12%	0	0%	>0.99
Dyspnea	11	42%	4	57%	0.67
Vertigo/syncope	4	15%	1	14%	>0.99
Peripheral edema	4	15%	3	43%	0.15
Myalgia	4	15%	1	14%	>0.99
Fatigue	17	65%	2	29%	0.11
Ptosis	1	4%	1	14%	0.39
Biological parameters during irAE (median, Q1-Q3)		n		n	
Peak hs-troponin T (<14 ng/L)	125 (89-290.5)	26	966 (250-1934)	7	**0.005 (∗∗)**
Peak hs-troponin I (<26 ng/L)	139 (34-259)	19	104 (71.75-398.8)	4	**0.97**
Peak NT-proBNP (<738 ng/L)	1,435 (625-3030)	23	9,174 (1,250-18,082)	7	**0.029 (∗)**
Peak D-dimer (<599 ng/mL)	2,170 (835.5-3,408)	14	3,748 (2,150-6,711)	5	0.087
Peak CK (25-190 U/L)	132 (78.5-347)	25	243 (106-524)	7	0.56
Peak CK-MB (<6% CK, U/L)	27 (14-63.75)	20	100 (22.25-245.3)	6	0.060
Peak AST (14-50 U/L)	39 (30.75-93.75)	26	150 (40-1,683)	7	**0.019 (∗)**
Peak ALT (11-60 U/L)	46 (26-90)	26	93 (69-3,133)	7	**0.026 (∗)**
Peak TSH (0.27-4.20 mU/L)	2.385 (1.145-4.678)	26	2.3 (0.7725-22.22)	5	0.62
Clinical findings					
Abnormal ECG, n	12	46%	7	100%	**0.013 (∗)**
Supraventricular extrasystoles	1	4%	1	14%	0.39
T-wave abnormalities	2	8%	0	0%	>0.99
Any-grade AV block	3	12%	4	57%	**0.023 (∗∗)**
High-grade AV block	0	0%	3	43%	**0.006 (∗)**
Broad arrhythmia	6	23%	7	100%	**0.001 (∗∗∗)**
Myocarditis-associated imaging					
Transthoracic echocardiography (n):	23	88%	7	100%	
LVEF (median, Q1-Q3)	61 (58-76)		56 (44-65)		0.20
Pericardial effusion	0	0%	1	14%	
CMR (n)	15	58%	4	57%	
CMR-LVEF (median, Q1-Q3)	62 (57-68)		55 (49-58)		**0.043 (∗)**
Myocarditis confirmed	2	13%	2	50%	
Myocarditis suggestive	5	33%	2	50%	
Pericardial effusion	4	27%	2	50%	
Myocarditis confirmed/suspected	7	47%	4	100%	
Patient presentation					
Time from ICI start to symptom onset in weeks (median, Q1-Q3)	11.5 (5-17.25)		6 (3-25)		0.24
Hospitalization	23	88%	7	100%	>0.99
Cardiac arrest	0	0%	2	29%	**0.040 (∗)**
Pharmacological management					
Corticoids	17	65%	6	86%	0.40
Tocilizumab	5	19%	3	43%	0.32
Infliximab	3	12%	1	14%	>0.99
Mycophenolate mofetil (MMF)	1	4%	1	14%	0.37
Other therapy	2	8%	1	14%	0.51

Clinical parameters and findings associated with ICI-My diagnosis in the full cohort (n = 33). Associations between characteristics and severity were evaluated using the Mann-Whitney *U* test or Fisher's exact test, as appropriate. Significant *P* values (*P* < 0.05) are shown in **bold**. ∗*P* < 0.05, ∗∗*P* < 0.01, and ∗∗∗*P* < 0.001.

ALT = alanine aminotransferase; AST = aspartate aminotransferase; AV = atrioventricular; CK = creatine kinase; CK-MB = CK myocardial band; CMR = cardiac magnetic resonance; ECG = electrocardiography; ICI-My = immune checkpoint inhibitor–associated myocarditis; irAE = immune-related adverse events; NT-proBNP = N-terminal pro–B-type natriuretic peptide; TSH = thyroid-stimulating hormone.

**Table 4:** Patients, Treatments, and Clinical Characteristics

	Nonsevere (n = 26)	% within cohort	Severe (n = 7)	% within cohort	*P*	Total (n = 33)	% within cohort
Demographics							
Age (median, Q1-Q3)	69 (62-75)	N/A	58 (52-68)	N/A	0.357	66 (57-75)	N/A
Sex, female	7	27%	1	14%	0.652	8	24%
BMI, kg/m^2^ (median, Q1-Q3)	24.8 (22.4-26.7)	N/A	24.5 (20.7-26.0)	N/A	0.532	24.7 (22.4-26.6)	N/A
Past smoker	6	23%	3	43%	0.358	9	27%
Active smoker	8	31%	1	14%	0.642	9	27%
PY among smokers (median, Q1-Q3)	50 (35-70)	N/A	20	N/A	N/A	50 (20-65)	N/A
PY reported	5	19%	1	14%	N/A	6	18%
Hypertension	11	42%	3	43%	>0.999	14	42%
Diabetes mellitus	5	19%	1	14%	>0.999	6	18%
Dyslipidemia	8	31%	0	0%	0.154	8	24%
CVA/TIA	1	4%	0	0%	>0.999	1	3%
CAD	2	8%	0	0%	>0.999	2	6%
Atrial fibrillation	2	8%	1	14%	0.524	3	9%
Hypothyroidism	5	19%	1	14%	>0.999	6	18%
Prior antineoplastic therapy							
Anthracyclines	0	0%	1	14%	0.212	1	3%
Nonanthracycline chemotherapy	11	42%	4	57%	0.674	15	45%
Prior ICI	9	35%	2	29%	>0.999	11	33%
Tyrosine kinase inhibitors	4	15%	1	14%	>0.999	5	15%
Intrapericardial bleomycin injection	1	4%	0	0%	>0.999	1	3%
Thoracic radiotherapy	4	15%	1	14%	>0.999	5	15%
ICI regimen							
Combination therapy	6	23%	3	43%	0.358	9	27%
Ipilimumab–nivolumab	5	19%	3	43%	0.320	8	24%
Pembrolizumab–quavonlimab	1	4%	0	0%	>0.999	1	3%
Monotherapy	20	77%	4	57%	0.358	24	73%
Pembrolizumab	12	46%	3	43%	>0.999	15	45%
Atezolizumab	3	12%	0	0%	>0.999	3	9%
Nivolumab	3	12%	0	0%	>0.999	3	9%
Cemiplimab	1	4%	0	0%	>0.999	1	3%
Durvalumab	1	4%	1	14%	0.385	2	6%
Cancer type							
Lung	9	35%	3	43%	0.686	12	36%
Melanoma	6	23%	2	29%	-	8	24%
Liver	2	8%	0	0%	-	2	6%
Kidney	2	8%	0	0%	-	2	6%
Head and neck	2	8%	0	0%	-	2	6%
Other	5	19%	2	29%	-	7	21%
Other irAEs							
At least one other irAE	23	88%	6	86%	>0.999	29	88%
Colitis	7	27%	1	14%	0.652	8	24%
Myositis	6	23%	1	14%	>0.999	7	21%
Hepatitis	5	19%	0	0%	0.559	5	15%
Cardiovascular adverse events (nonmyocarditis)	4	15%	1	14%	>0.999	5	15%
Thyroiditis	3	12%	1	14%	>0.999	4	12%
Pneumonitis	2	8%	1	14%	0.524	3	9%
Pancreatitis	2	8%	0	0%	>0.999	2	6%
Hematological disorders	2	8%	1	14%	0.524	3	9%
Arthritis	2	8%	0	0%	>0.999	2	6%
SIADH (Syndrome of Inappropriate Antidiuretic Hormone Secretion)	2	8%	0	0%	>0.999	2	6%
Other unique irAEs	4	15%	0	0%	N/A	4	12%

Clinical, demographic, and therapeutic characteristics of the full cohort (n = 33). Associations between characteristics and severity were evaluated using the Mann-Whitney *U* test or Fisher's exact test, as appropriate. Significant *P* values (*P* < 0.05) are shown in **bold**. ∗*P* < 0.05, ∗∗*P* < 0.01, and ∗∗∗*P* < 0.001.

BMI = body mass index; CAD = coronary artery disease; CVA= cerebral vascular accident;; ICI = immune checkpoint inhibitor; irAE = immune-related adverse events; PY = pack years; TIA = transient ischemic attack.

### Baseline characteristics of patients with ICI-My

Baseline characteristics are summarized in Table 4. Median body mass index was similar between the nonsevere and severe groups (24.8 [Q1–Q3: 22.4-26.7] vs 24.5 [Q1–Q3: 20.7-26.0] kg/m^2^). Cardiovascular risk factors were frequent, including hypertension in 14 of 33 patients (42%), diabetes in 6 of 33 (18%), and dyslipidemia in 8 of 33 (24%); established cardiovascular disease was uncommon (coronary artery disease, 2 of 33 [6%]; atrial fibrillation, 3 of 33 [9%]). The most common cancers were lung cancer (12 of 33, 36%) and melanoma (8 of 33, 24%), followed by liver, kidney, and head and neck cancers (2 of 33 each, 6%) and other tumors (7 of 33, 21%). Most patients received ICI monotherapy (24 of 33, 73%) rather than combination therapy (9 of 33, 27%), with pembrolizumab being the most frequent regimen (15 of 33, 45%) and ipilimumab–nivolumab accounting for most combinations (8 of 33, 24%) (Table 4). Prior anticancer therapies were common (nonanthracycline chemotherapy, 15 of 33 [45%]; prior ICI exposure, 11 of 33 [33%]; thoracic radiotherapy, 5 of 33 [15%]), whereas anthracycline exposure was rare (1 of 33, 3%). Concomitant irAEs were frequent, with at least one additional irAE in 29 of 33 patients (88%), most often colitis (8 of 33, 24%) and myositis (7 of 33, 21%) (Table 2). No demographic characteristics differed significantly between severity groups.

### Clinical presentation of patients with ICI-My

Clinical presentation is detailed in Table 3. The most common symptoms were fatigue (19 of 33, 58%), dyspnea (15 of 33, 45%), pain (13 of 33, 39%), and chest pain (12 of 33, 36%); syncope or vertigo occurred in 5 of 33 (15%), and peripheral edema occurred in 7 of 33 (21%). Electrocardiographic abnormalities were present in 19 of 33 patients (58%), including any-degree atrioventricular (AV) block in 7 of 33 (21%) and high-grade AV block in 3 of 33 (9%); a broader category of clinically relevant rhythm or conduction disturbances (“broad arrhythmia”) was observed in 13 of 33 (39%) (Table 3). Transthoracic echocardiography was performed in 30 of 33 patients (91%) (Table 3). CMR imaging was obtained in 19 of 33 patients (58%). CMR findings were confirmatory in 4 of 19 (21%; 12% of the total cohort), suggestive in 7 of 19 (37%; 21% of the total cohort), and negative in 8 of 19 (42%; 24% of the total cohort), and CMR was not performed in 14 of 33 patients (42%) (Tables 2 and 3).

All seven severe patients and 23 of 26 nonsevere patients were hospitalized (30 of 33, 91%), and cardiac arrest occurred in 2 of 33 patients (6%) (Table 3). Immunosuppression most commonly included corticosteroids (23 of 33, 70%), with escalation to TCZ (8 of 33, 24%), infliximab (4 of 33, 12%), or mycophenolate mofetil (2 of 33, 6%) in selected cases (Table 3).

### Clinical presentation of patients with severe vs nonsevere ICI-My

Marked differences emerged when patients were stratified by IC-OS severity (Tables 2, 3, and 4). Severe ICI-My was characterized by higher cardiac injury and hemodynamic stress markers, including higher peak hs-troponin T (median 966 [Q1–Q3: 250-1934] vs 125 [Q1–Q3: 89-290.5] ng/L; *P* = 0.005) and peak NT-proBNP (median 9,174 [Q1–Q3: 1,250-18,082] vs 1,435 [Q1–Q3: 625-3,030] ng/L; *P* = 0.029). Severe cases also had higher transaminase levels (aspartate aminotransferase [AST]: median 150 [Q1–Q3: 40-1,683] vs 39 [Q1–Q3: 30.75-93.75] U/L; *P* = 0.019; alanine aminotransferase [ALT]: median 93 [Q1–Q3: 69-3,133] vs 46 [Q1–Q3: 26-90] U/L; *P* = 0.026) (Table 3).

Electrical instability was a defining feature: Electrocardiographic abnormalities were present in 100% of severe patients vs 46% of nonsevere patients (7 of 7 vs 12 of 26; *P* = 0.002); any AV block occurred in 57% vs 12% (4 of 7 vs 3 of 26; *P* = 0.023), and high-grade AV block occurred in 43% vs 0% (3 of 7 vs 0 of 26; *P* = 0.006). “Broad arrhythmia” was observed in 100% vs 23% (7 of 7 vs 6 of 26; *P* < 0.001) (Table 3), consistent with Table 2 findings showing ventricular arrhythmia and/or conduction system disease in 86% of severe patients vs 15% of nonsevere patients (6 of 7 vs 4 of 26; *P* = 0.019). Functional impairment also differed: Decline in systolic function was present in 100% of severe patients vs 31% of nonsevere patients (7 of 7 vs 8 of 26; *P* = 0.002) (Table 2), and CMR-derived left ventricular ejection fraction was lower in severe cases (median 55% [Q1–Q3: 49% to 58%] vs 62% [Q1–Q3: 57% to 68%]; *P* = 0.043) (Table 3). By IC-OS determinants, severe myocarditis was driven by hemodynamic instability (3 of 7, 43%), heart failure requiring ventilatory support (1 of 7, 14%), complete or high-grade heart block (3 of 7, 43%), and significant ventricular arrhythmia (2 of 7, 29%), all of which were absent in nonsevere cases (Table 2). Clinically, cardiac arrest occurred exclusively in severe myocarditis (29% [2 of 7] vs 0% [0 of 26]; *P* = 0.040) (Table 3).

### Comprehensive cytokine and biological profiles in ICI-My

**Figure 1:**
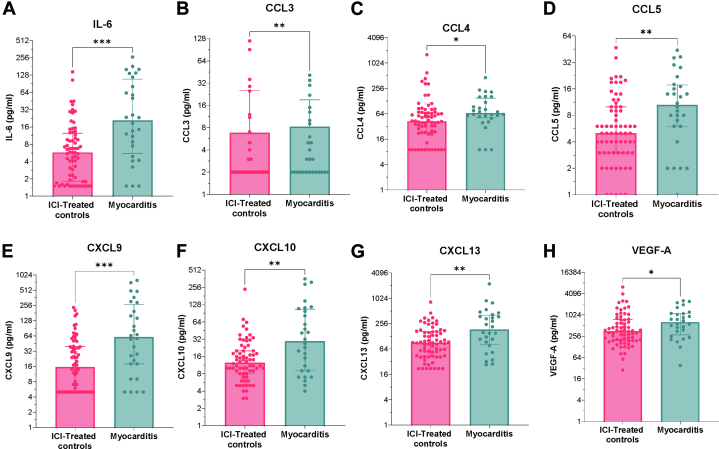
Cytokine Profile of ICI-My vs ICI-Treated Controls (A–H) Serum levels of various chemokines, growth factors, and cytokines measured at ICI-My diagnosis in 28 patients were compared with serum levels from 68 ICI-treated control patients. Comparisons were performed using the Mann-Whitney *U* test. Data are presented as individual values overlaid on box plots; center lines indicate medians, and boxes indicate 25th-75th percentiles. Statistical significance is indicated as ∗*P* < 0.05, ∗∗*P* < 0.01, and ∗∗∗*P* < 0.001. ICI-My: immune checkpoint inhibitor–associated myocarditis.

Multiplex cytokine profiling revealed higher circulating levels of IL-6, CCL3, CCL4, CCL5, CXCL9, CXCL10, CXCL13, and VEGF-A in patients with ICI-My than in ICI-treated control patients (Figure 1). The ICI-treated control cohort comprised 68 patients, with demographic characteristics detailed in Supplemental Table 1. After Benjamini–Hochberg correction, the main cytokine differences remained significant at q < 0.01 (Supplemental Figure 3A).

**Figure 2:**
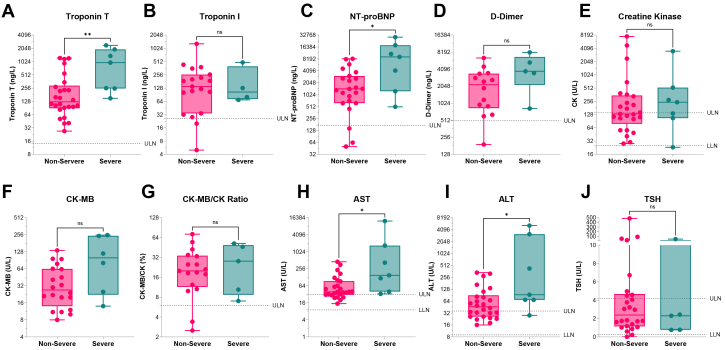
Conventional Biomarkers According to ICI-My Severity (A–J) Biomarker levels were compared between patients with severe ICI-My (n = 7) and nonsevere ICI-My (n = 26). (A) Troponin T (n = 33), (B) Troponin I (n = 23), (C) NT-proBNP (n = 30), (D) D-dimer (n = 19), (E) total CK (n = 32), (F) CK-MB (n = 26), (G) CK-MB/CK ratio (n = 22), (H) AST (n = 33), (I) ALT (n = 33), and (J) TSH (n = 31). Each plot shows individual patient values overlaid on box plots; center lines indicate medians, and boxes indicate 26th-75th percentiles. Comparisons were performed using the Mann-Whitney *U* test. Statistical significance is indicated as ∗*P* < 0.05, ∗∗*P* < 0.01, and ns = not significant. ULN and LLN denote institutional upper and lower limits of normal, respectively; LLN was available only for CK and TSH. ALT = alanine aminotransferase; AST = aspartate aminotransferase; ICI-My = immune checkpoint inhibitor–associated myocarditis; NT-proBNP = N-terminal pro–B-type natriuretic peptide; cTnT = cardiac troponin T; cTnI = cardiac troponin I; CK = creatine kinase; MB = myocardial band; TSH = thyroid-stimulating hormone; ULN = upper limit of normal; LLN = lower limit of normal.

To further characterize ICI-My, we analyzed an extensive panel of cardiac injury and hemodynamic stress biomarkers, including troponin T (cTnT), troponin I, NT-proBNP, CK, CK-MB, and the CK-MB/CK ratio, as well as selected systemic biomarkers, including D-dimer, thyroid-stimulating hormone, AST, and ALT. Among these, cTnT, NT-proBNP, AST, and ALT were higher in severe cases than in nonsevere cases (Figure 2). After Benjamini–Hochberg correction, troponin T, NT-proBNP, AST, and ALT remained significant at q < 0.05 (Supplemental Figure 3B). In contrast, no single cytokine consistently distinguished severe from nonsevere myocarditis (Supplemental Figure 4). Exploratory correlation analysis revealed several notable associations: CK correlated positively with cTnT, AST, and ALT, and negatively with CK-MB; AST and ALT correlated with CCL2 and CK; and NT-proBNP correlated with IL-6 (Supplemental Figure 5).

### Characterization of immune cell subset distribution in ICI-My

**Figure 3:**
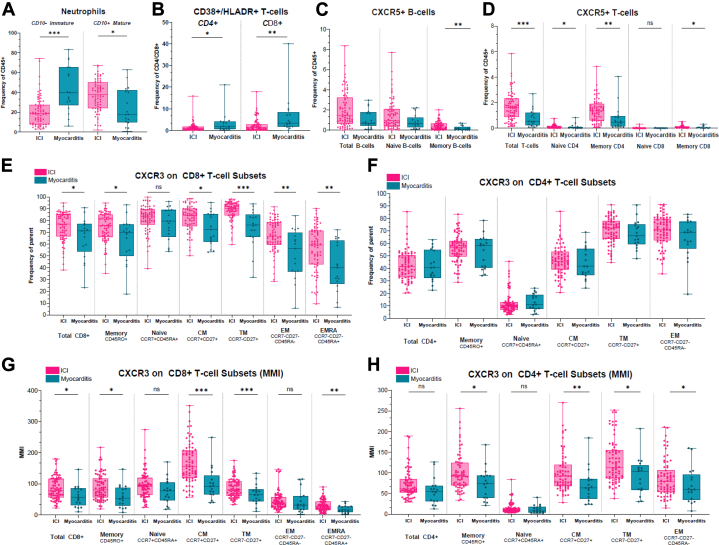
Distribution and Phenotypes of Circulating Immune Cell Populations in ICI-My (A) Frequencies of circulating immature (CD10^–^) and mature (CD10^+^) neutrophils, expressed as percentages of CD45^+^ cells. (B) Frequencies of activated T cells (CD38^+^HLA-DR^+^) within CD4^+^ and CD8^+^ T-cell subsets. (C) Frequencies of CXCR5^+^ B-cell subsets and (D) CXCR5^+^ T-cell subsets. (E and F) Frequencies of CXCR3^+^ cells within CD8^+^ and CD4^+^ T-cell subsets, respectively; (G and H) MMI of CXCR3 expression within CD8^+^ and CD4^+^ T-cell subsets, respectively. Data were obtained from 16 patients at ICI-My diagnosis and compared with data from 72 cancer patients treated with ICI but without myocarditis or other irAEs, as detailed in Supplemental Table 1. Statistical significance was determined using the Mann-Whitney *U* test, with ∗*P* < 0.05, ∗∗*P* < 0.01, ∗∗∗*P* < 0.001, and ns = not significant. ICI-My = immune checkpoint inhibitor–associated myocarditis; irAEs = immune-related adverse events; MMI = mean metal intensity.

Mass cytometry analyses compared patients with ICI-My with ICI-treated patients without myocarditis or other irAEs (n = 72; Supplemental Table 1), thereby controlling more directly for immune changes related to ICI exposure itself. ICI-My was associated with a higher proportion of immature neutrophils, increased activated HLA-DR^+^CD38^+^ CD4^+^ and CD8^+^ T cells, and lower CXCR5^+^ memory B- and T-cell populations (Figure 3). The observed shifts in neutrophil maturity suggest an acute inflammatory response, whereas the increase in HLA-DR^+^CD38^+^ T cells indicates heightened T-cell activation. These changes were accompanied by contraction of switched and unswitched memory B-cell compartments and reduced frequencies of CXCR3-expressing CD8^+^ T cells, particularly within central memory, effector memory, and terminal memory subsets, and lower CXCR3 expression across memory T-cell subsets in both the CD4^+^ and CD8^+^ compartments (Figure 3 and Supplemental Figure 6).

No notable differences were observed in the frequencies of the three monocyte subsets (classical, intermediate, and nonclassical) or in mean metal intensity for activation and functional markers (CD11c, HLA-DR, CD141, CD31, CD38, CD123, CD62L, CD69, and CD1c), except for nonclassical monocytes, which showed minor but significant decreases in activation-marker expression in patients with myocarditis (Supplemental Figure 6). Because these analyses are exploratory and based on a limited number of myocarditis samples, they should be interpreted as immune correlates rather than definitive diagnostic markers.

### Evaluation of complement system involvement in ICI-My

To explore complement involvement, we assessed markers spanning the classical, alternative, and terminal pathways. Plasma C4, CH50, C3c, factor Bb, and soluble C5b-9 did not show a consistent systemic activation signal in this cohort (Supplemental Figure 7). These negative plasma findings do not exclude tissue-level complement deposition within the myocardium.

### Potential role of TCZ in corticosteroid-refractory ICI-My

**Figure 4:**
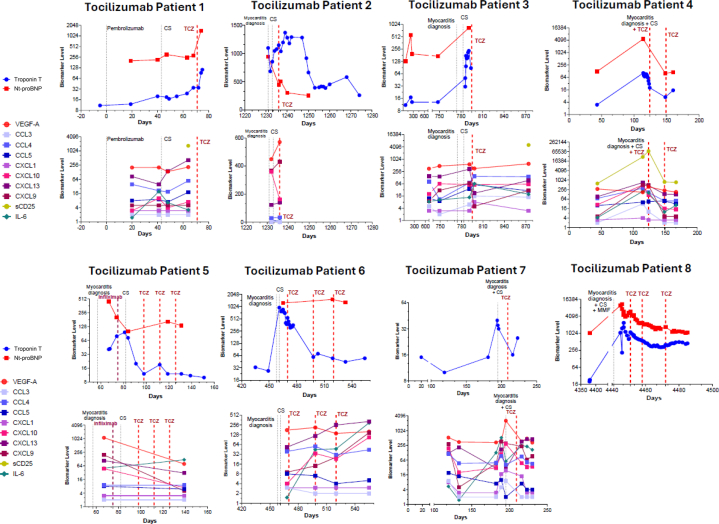
Longitudinal Biomarker Trajectories After TCZ in CS-Refractory ICI-My Each panel represents an individual patient (n = 8) treated with TCZ after inadequate response to CS therapy. Longitudinal changes in cardiac biomarkers (cTnT and NT-proBNP, ng/L) and selected cytokines and chemokines are shown over time. Myocarditis diagnosis and the timing of immunosuppressive treatments are indicated by labeled dashed vertical lines on each patient's panel. Some time points are missing because assessments were not performed. CS = corticosteroids; TCZ = tocilizumab; NT-proBNP = N-terminal pro–B-type natriuretic peptide; cTnT = cardiac troponin T; ICI-My = immune checkpoint inhibitor–associated myocarditis; MMF = mycophenolate mofetil.

Because IL-6 was among the most consistently elevated circulating mediators, and given our prior report of a corticosteroid-refractory ICI-My case successfully managed with TCZ,^10^ we examined a real-world series of eight patients with corticosteroid-refractory or less-responsive ICI-My treated with TCZ on a compassionate basis. The clinical characteristics of this cohort are presented in Supplemental Table 2. Melanoma (3 of 8, 38%) and lung cancer (2 of 8, 25%) were the most prevalent tumor types; six of eight patients (75%) received combination anti-CTLA-4/anti-PD-1 regimens, and two of eight (25%) received anti-PD-1 therapy alone. Colitis was the most common concurrent irAE, occurring in four of eight patients (50%). At initial presentation, three of eight patients (38%) had severe myocarditis. TCZ was introduced after multidisciplinary review, usually in addition to corticosteroids and, in selected cases, other immunosuppressive agents. Biomarker trajectories after TCZ were generally favorable in most patients, although responses were heterogeneous, and interpretation is limited by nonstandardized sampling and possible selection bias (Figure 4 and Supplemental Figure 8). Seven patients survived the acute episode. The eighth patient (patient 1 in Figure 4) made the decision to withdraw from all treatment and ultimately died. Corticosteroids were successfully tapered in the 7 patients who survived. Four of eight patients (50%) required only a single TCZ dose.

## Discussion

Our study provides an integrated peripheral immune portrait of ICI-My by combining multiplex cytokine profiling, mass cytometry analyses in ICI-treated control patients without myocarditis, conventional cardiac biomarkers, and a small observational TCZ series. The main finding is that ICI-My is associated with an IL-6–centered and chemokine-centered inflammatory signature that remains detectable when compared with patients exposed to ICIs but without myocarditis (Central Illustration). This strengthens the biological relevance of the observed immune differences beyond nonspecific immune activation induced by ICI therapy alone. The cytokine profile observed here—marked by IL-6, CXCL9, CXCL10, CXCL13, and inflammatory chemokines—is consistent with prior reports implicating interferon-γ–linked inflammation and myeloid activation in ICI-My and other severe immune toxicities.^10^^,^^19^, ^20^, ^21^, ^22^, ^23^

At the cellular level, mass cytometry analysis indicates that ICI-My is associated with activated T-cell states, myeloid immaturity, disruption of CXCR5^+^ and memory B-cell compartments, and reduced frequencies of CXCR3^+^-expressing CD8^+^ T cells and lower CXCR3 expression across memory T-cell subsets in both the CD4^+^ and CD8^+^ compartments. Recent work using a mouse model of PD-1 deficiency–associated myocarditis identified CXCR3-dependent recruitment of effector CD8^+^ T cells as a mechanism contributing to cardiomyocyte injury.^24^ These findings are biologically consistent with our results, notably the increased circulating levels of the CXCR3 ligands CXCL9 and CXCL10 together with the reduced frequency of circulating CXCR3^+^ CD8^+^ T cells, which may reflect preferential trafficking of these cells toward inflamed myocardial tissue. Although these observations remain peripheral correlates rather than direct surrogates of myocardial tissue biology, they support the concept that ICI-My reflects a systemic inflammatory program rather than isolated cardiac biomarker elevation. At the same time, our data indicate that conventional biomarkers remain more informative than individual cytokines for severity assessment in this cohort. As previously reported,^25^^,^^26^ hs-troponin T, NT-proBNP, AST, and ALT distinguished severe from nonsevere presentations, whereas circulating cytokines did not. This distinction is clinically important: Immune profiling may help refine pathobiology and identify candidate pathways, but current bedside risk stratification still relies primarily on markers of myocardial injury, wall stress, and electrical instability.

Our results are also broadly aligned with larger prognostic studies, including the recently proposed score for ICI-My,^26^ in which incremental troponin elevation (20- to 2,000-fold above the upper reference limit) emerged as one of the strongest predictors of 30-day major cardiomyotoxic events, alongside left ventricular dysfunction, low QRS voltage, cardiomuscular symptoms, and thymoma. Together with data from previous reports,^26^^,^^27^ our results support the view that troponin and other markers of myocardial injury and wall stress remain the backbone of severity assessment in ICI-My, whereas multiparametric immune profiling may provide complementary information in selected scenarios, particularly at the extremes of risk or when escalation to targeted immunomodulation, such as IL-6R blockade, is being considered. This concept requires validation in larger prospective studies.

Recent reports have suggested a potential role for complement activation in ICI-My;^28^^,^^29^ however, in our cohort, systemic markers spanning the classical, alternative, and terminal pathways (C4, CH50, C3c, factor Bb, and sC5b-9) did not show a clear activation signal. This negative systemic finding may reflect differences in sampling time relative to the acute phase, a predominance of local myocardial complement deposition rather than systemic activation, or heterogeneity in case mix and assay platforms. Importantly, the absence of systemic activation does not exclude tissue-level complement involvement. Dedicated studies integrating plasma split products with myocardial immunostaining and temporal sampling are needed to clarify the contribution of complement in ICI-My.

The observed inflammatory profile provides mechanistic support for evaluating IL-6R blockade in selected corticosteroid-refractory cases, consistent with our prior report.^10^ However, the TCZ data should be interpreted cautiously. This was a small, uncontrolled case series with nonstandardized treatment timing, concomitant immunosuppressive therapies, and outcome ascertainment limited by the retrospective design. Accordingly, our findings support feasibility and biological plausibility, not comparative efficacy or a new standard of care. More broadly, IL-6R blockade may have therapeutic relevance across selected severe irAEs, as we have previously reported in cholangio-hepatitis,^9^ cytokine release syndrome (CRS),^8^ immune-related hemophagocytic lymphohistiocytosis (irHLH),^8^^,^^30^ esophageal stenosis,^31^ and arthritis.^32^

This study has several limitations. First, the single-center retrospective design and limited sample size—particularly for the severe myocarditis, mass cytometry, and TCZ subgroups—restrict precision and generalizability. Second, although the control cohorts are more biologically appropriate than untreated cancer controls, they remain heterogeneous with respect to tumor type, ICI regimen, and time from ICI initiation to sampling. Residual confounding cannot be excluded. Third, cytokine sampling, imaging, and treatment timing were not standardized, and batch effects may have occurred in multiplex cytokine analyses. Fourth, the inclusion of nonsevere, often smoldering or asymptomatic myocarditis identified through systematic troponin screening has both strengths and limitations: It enhances sensitivity and captures the full clinical spectrum, but it also complicates comparisons with historical cohorts restricted to fulminant cases and raises unresolved questions about long-term prognosis. Our follow-up duration was insufficient to robustly evaluate late cardiomyopathy or arrhythmic events, particularly in nonsevere disease, and this remains a key priority for future studies.

In conclusion, ICI-My is associated with a distinct peripheral inflammatory signature centered on IL-6 and interferon-γ–inducible chemokines, even when compared with ICI-treated control patients without myocarditis. Peripheral blood mass cytometry analyses show shifts in activated T cells, immature neutrophils, and memory B-cell compartments. Conventional cardiac biomarkers remain the strongest bedside indicators of severity in this cohort, whereas IL-6R blockade emerges as a biologically plausible strategy for selected steroid-refractory cases that should be tested prospectively.

Perspectives **COMPETENCY IN MEDICAL KNOWLEDGE:** Immune checkpoint inhibitor (ICI)-associated myocarditis is characterized by a systemic IL-6–centered and chemokine-centered inflammatory signature that remains detectable against the background immune effects of ICI therapy when biologically appropriate ICI-treated controls are used. In contrast, no clear systemic complement activation signal was identified in this cohort, suggesting that circulating complement biomarkers may have limited clinical utility for routine blood-based assessment, although localized or temporally restricted complement involvement cannot be excluded. In current practice, severity assessment continues to rely mainly on conventional markers of myocardial injury, hemodynamic stress, and electrical instability.

**TRANSLATIONAL OUTLOOK:** Prospective multicenter studies are needed to determine whether cytokine profiling and peripheral immune phenotyping provide clinically meaningful incremental value for diagnosis or early risk stratification beyond troponin, natriuretic peptides, electrocardiography, and cardiac imaging and to clarify whether complement activation is absent systemically or confined to selected biological compartments or disease subsets. Selected steroid-refractory cases of immune checkpoint inhibitor–associated myocarditis provide translational rationale for prospective controlled evaluation of IL-6 receptor blockade using standardized escalation criteria, serial biospecimen collection, and predefined clinical and biomarker end points.

## Funding Support and Author Disclosures

Dr Obeid has received honoraria and speaker fees with Moderna, Roche, and BMS. Dr Peters has received honoraria with Roche, Bristol Myers Squibb, Novartis, Pfizer, MSD, AstraZeneca, Takeda, and Illumina and consulting or advisory role with Roche/Genentech, Novartis, Bristol Myers Squibb, Pfizer, MSD, Amgen, AstraZeneca, Janssen, Regeneron, and Merck Serono. Dr Boughdad receives honoraria and speaker fees from Novartis. All other authors have reported that they have no relationships relevant to the contents of this paper to disclose.
