# Supplementary material for: Immune Profiling Identifies Inflammatory Signatures in Immune Checkpoint Inhibitor–Related Myocarditis
Source: JACC CardioOncol. 2026 Jun 16;8(3):245–62. doi: 10.1016/j.jaccao.2026.04.008 (PMC13282819; doi:10.1016/j.jaccao.2026.04.008)
Supplement: Supplemental Figures 1 to 9 and Supplemental Tables 1 to 4 [file mmc1.docx]

**Immune Profiling Identifies Inflammatory Signatures in Checkpoint Inhibitor-Related Myocarditis**

Douglas Daoudlarian^≠a^ PhD, Sarah Boughdad^b≠^ MD, Robin Bartolini^a^ PhD, Sofiya Latifyan^c^ MD, Jacqueline Doms^a^ MD, Hasna Bouchaab^c^ MD, Karim Abdelhamid^c^ MD, Nabila Ferahta^c^ MD, Nuria Neisy Mederos Alfonso^c^ MD, Victor Joo^a^ PhD, Antonia Stamatiou^c^ MD, Lucrezia Mencarelli^a^ MD, Nicolas Etienne^b^ MD, Athina Stravodimou^c^ MD, Khalil Zaman^c^ MD, Matthieu Perreau^a^ PhD, Craig Fenwick^a^ PhD, Keyvan Shabafrouz^c^ MD, Giuseppe Pantaleo^a^ MD, Solange Peters^c^ MD-PhD, Michel Obeid^a*^ MD-PhD

^a^Centre Hospitalier Universitaire Vaudois (CHUV), University of Lausanne, Department of Medicine, Immunology and Allergy Service, Rue du Bugnon 46, CH-1011 Lausanne, Switzerland

^b^Nuclear Medicine Department, Groupe Hospitalier Pitié-Salpêtrière, Assistance Publique-Hôpitaux de Paris (AP-HP), Sorbonne Université, Paris, France.

^c^Centre Hospitalier Universitaire Vaudois (CHUV), University of Lausanne, Department of Oncology, Medical Oncology Service, Rue du Bugnon 46, CH-1011 Lausanne, Switzerland

≠contributed equally to this work

*** Correspondence:** Pr Michel Obeid, MD-PhD

Lausanne Center for Immuno-Oncology Toxicities LCIT

Immunology and Allergy Division, Rue du Bugnon 17, 1011 Lausanne, Switzerland

Centre Hospitalier Universitaire Vaudois (CHUV)

**Email:** [michel.obeid@chuv.ch](mailto:michel.obeid@chuv.ch) - **Phone :** +41 (0)21 314 07 90

**Supplemental** methods

Immune profiling of circulating blood immune-cell populations by mass cytometry. Peripheral blood immune-cell profiling by mass cytometry was performed as previously described Briefly, cells were stained with a metal-conjugated antibody cocktail, fixed with paraformaldehyde (PFA), lysed, and incubated overnight at 4°C with additional metal-conjugated antibodies and Cell-ID Intercalator. Data acquisition was performed on a HELIOS CyTOF system and normalized using EQ Four Element Calibration Beads. For the revised analyses, immune-cell distributions in 16 patients with ICI-My at diagnosis were compared with those in 72 ICI-treated cancer controls without myocarditis or other clinically apparent irAEs. The 46-antibody panel and gating strategy are detailed in Supplemental Figure 1.

**Immune profiling of serum cytokines.** As previously described(2-7), Cytokine measurements were performed in the routine diagnostic workflow of our Immunology laboratory, which analyzes cytokines on a daily basis in patients with cancer, with or without ICI exposure. Samples from ICI-myocarditis cases and ICI-treated controls (n=68) were processed using the same standardized assay platform, the same operating protocol, the same technical staff, and the same routine laboratory procedures. We quantified 49 cytokines, chemokines, and growth factors using the Luminex ProcartaPlex immunoassay, with IL-6 measured on the Cobas platform. A list of biomarkers, lower limits of detection, and descriptive distributions within the cohort is provided in Supplemental Table 3. Sample values at or below the lower limit of detection (LLOD) were replaced by the LLOD. We analyzed cytokine levels at myocarditis onset, before immunosuppression, and during subsequent treatments for longitudinal analyses. A cohort of 68 ICI-treated patients who had received at least 2 ICI cycles without myocarditis or other clinically apparent immune-related adverse events (irAEs) served as the main cytokine control group. This design was intended to distinguish myocarditis-associated inflammatory changes from background immune activation related to ICI exposure alone.

**Supplemental figures and tables**


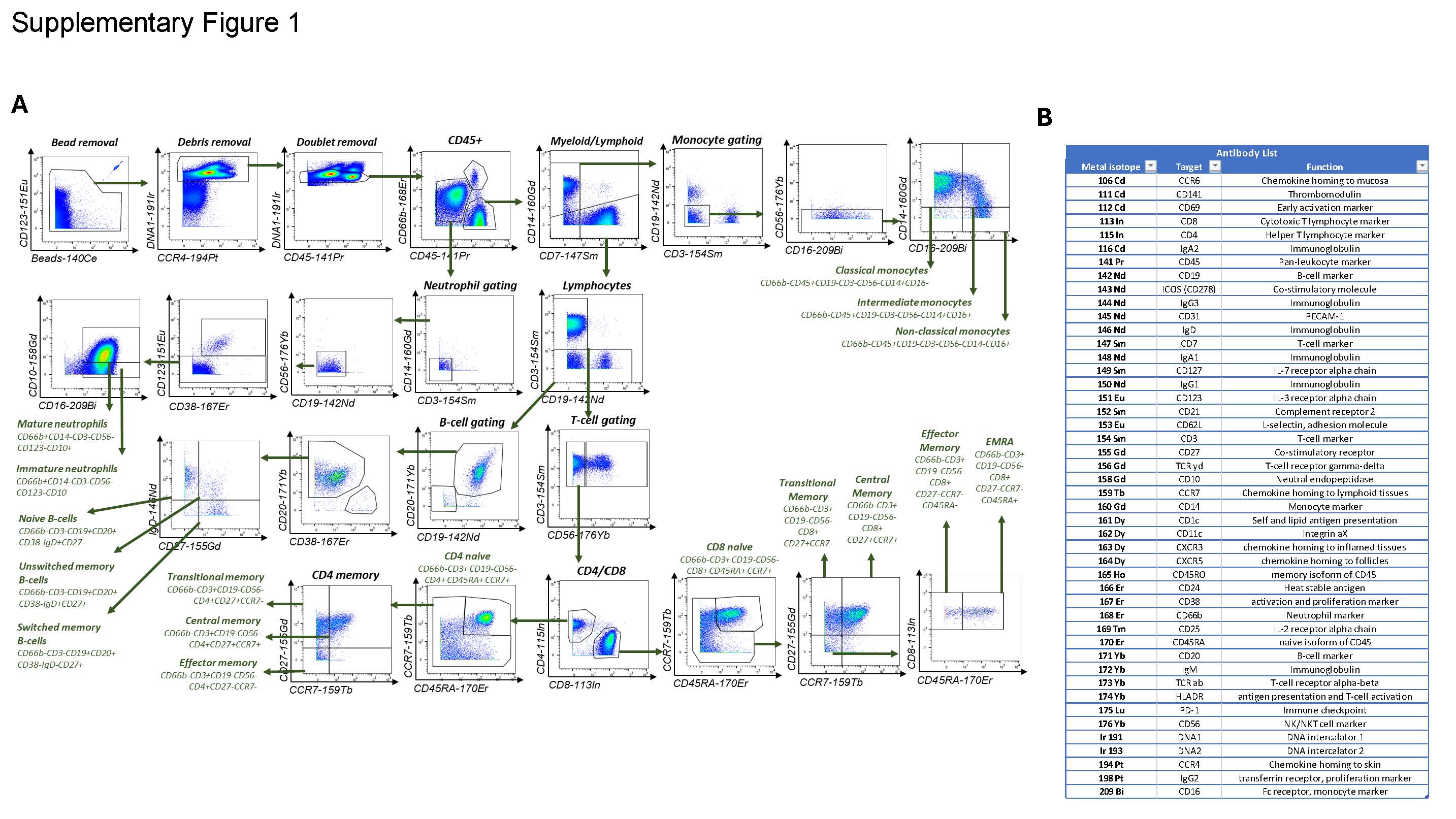
Supplemental Figure 1: Gating strategy for mass cytometry. Complete gating strategy used to identify the main immune-cell populations, together with the list of antibodies and corresponding metal conjugates.


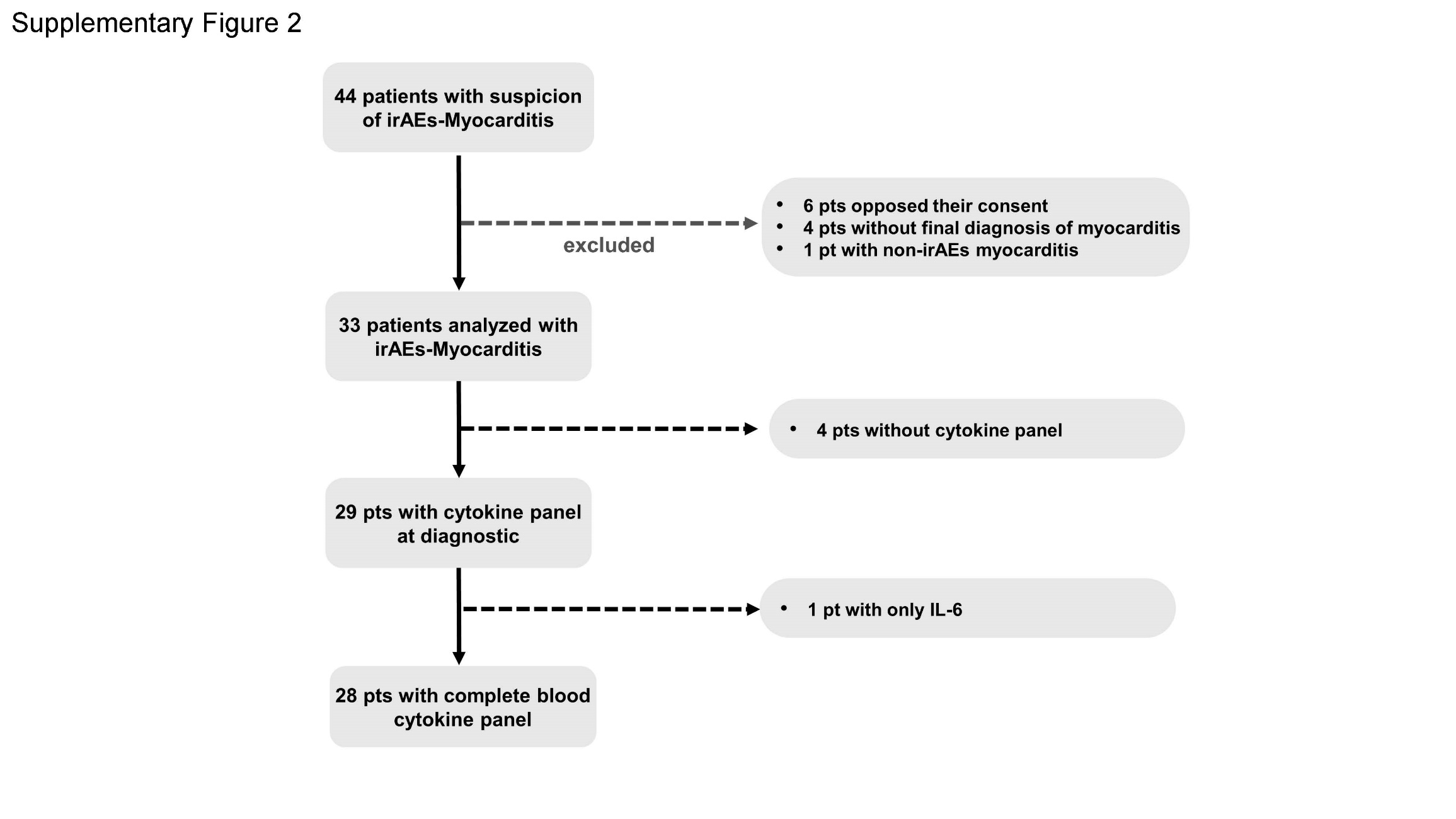
Supplemental Figure 2: Study flowchart


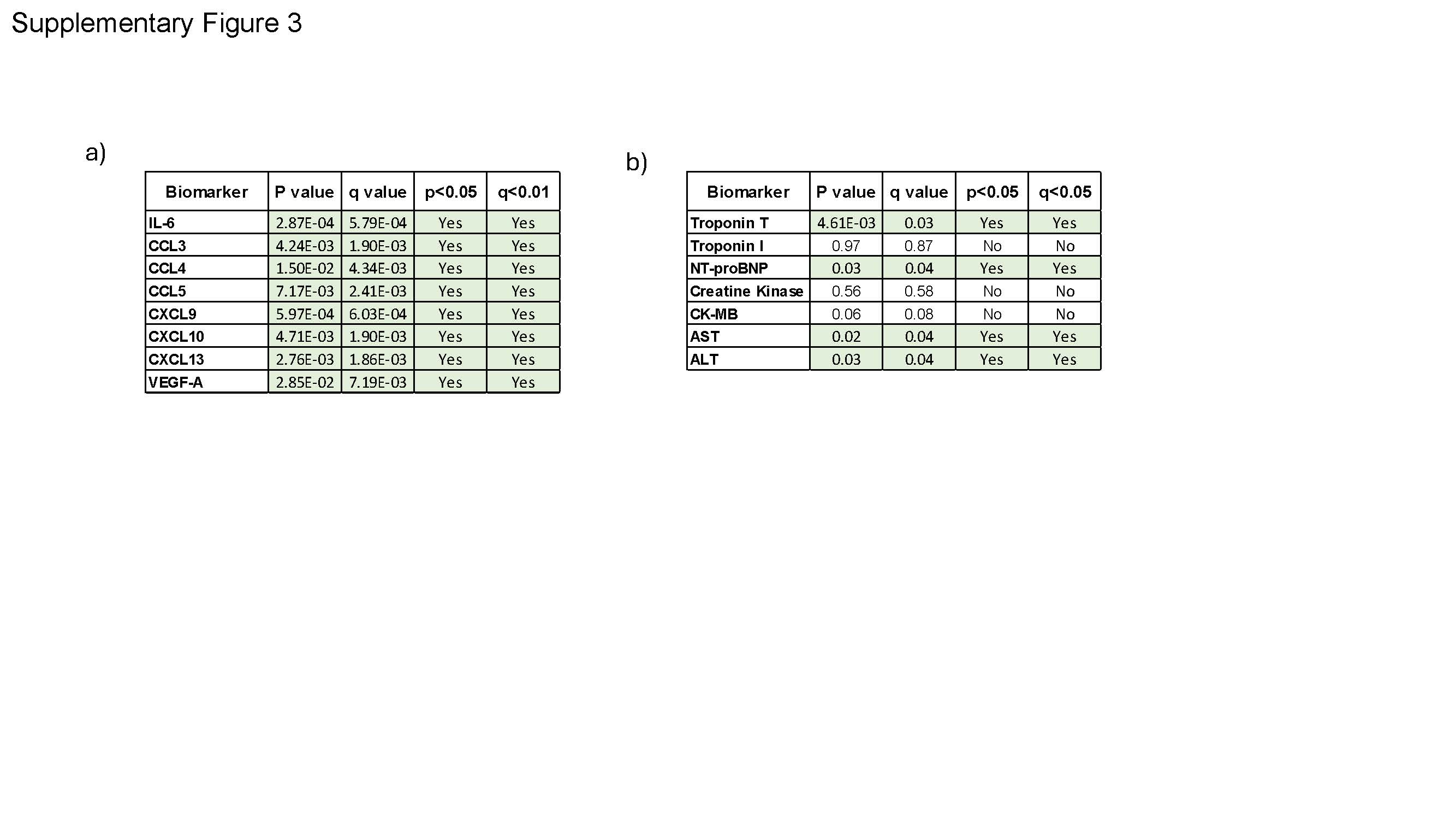
Supplemental Figure 3. False discovery rate correction analyses. (A-B) False discovery rate (FDR) analyses for comparisons of cytokines between ICI-treated controls and patients with ICI-My (A), and of cardiac-associated biomarkers between non-severe and severe ICI-My (B), using the Benjamini-Hochberg method. The corresponding desired q values were 0.01 and 0.05, respectively.

**
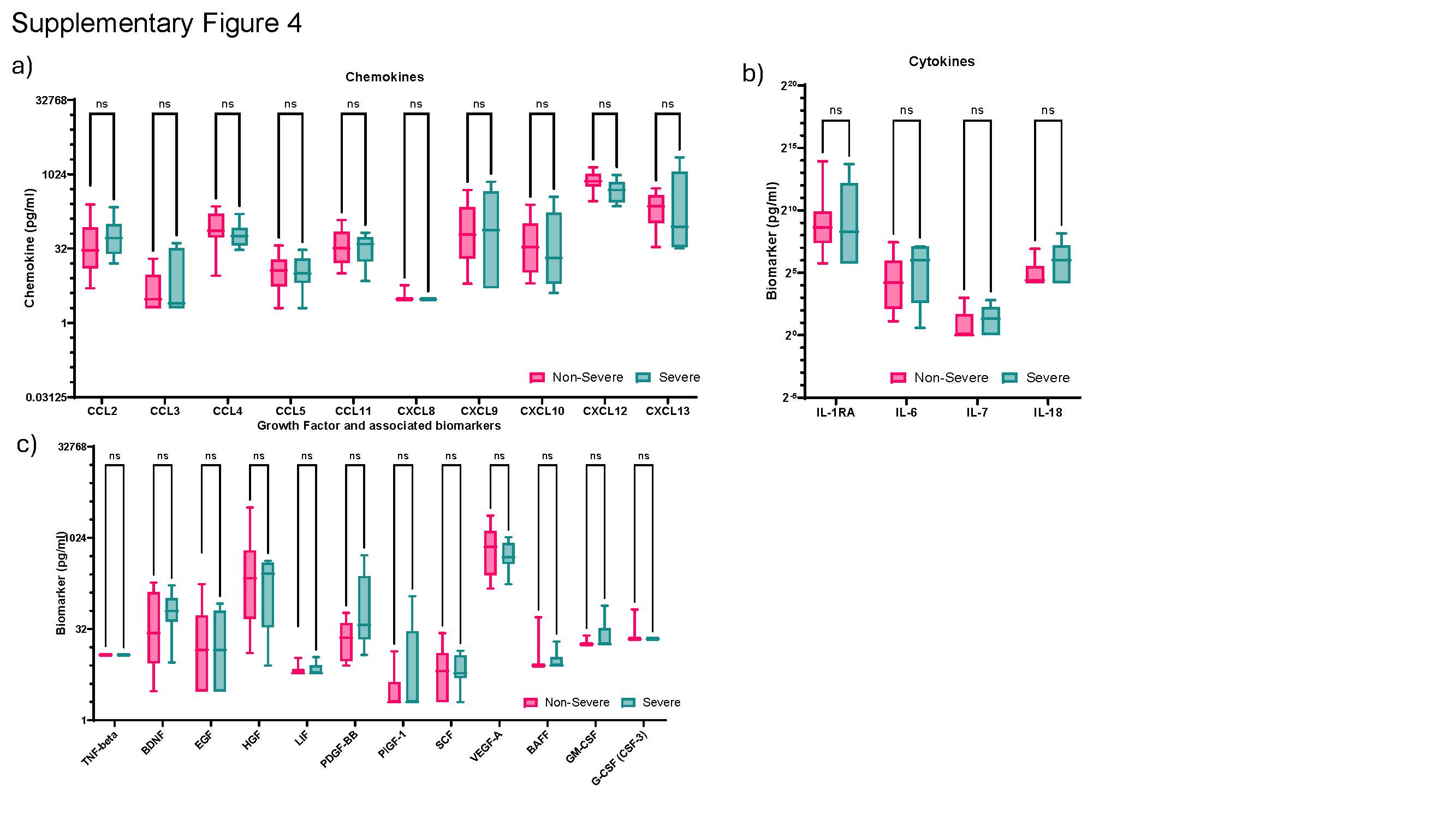
**

****Supplemental Figure 4.**** Cytokine profiles according to myocarditis severity. Serum levels of chemokines (A), cytokines (B), and growth factors (C) measured at ICI-My diagnosis in 28 patients, stratified by non-severe versus severe ICI-My. All comparisons were performed using the Mann-Whitney U test. Data are presented as individual values, with bars indicating median levels and error bars representing the interquartile range (IQR), 10-90 percentiles.

**
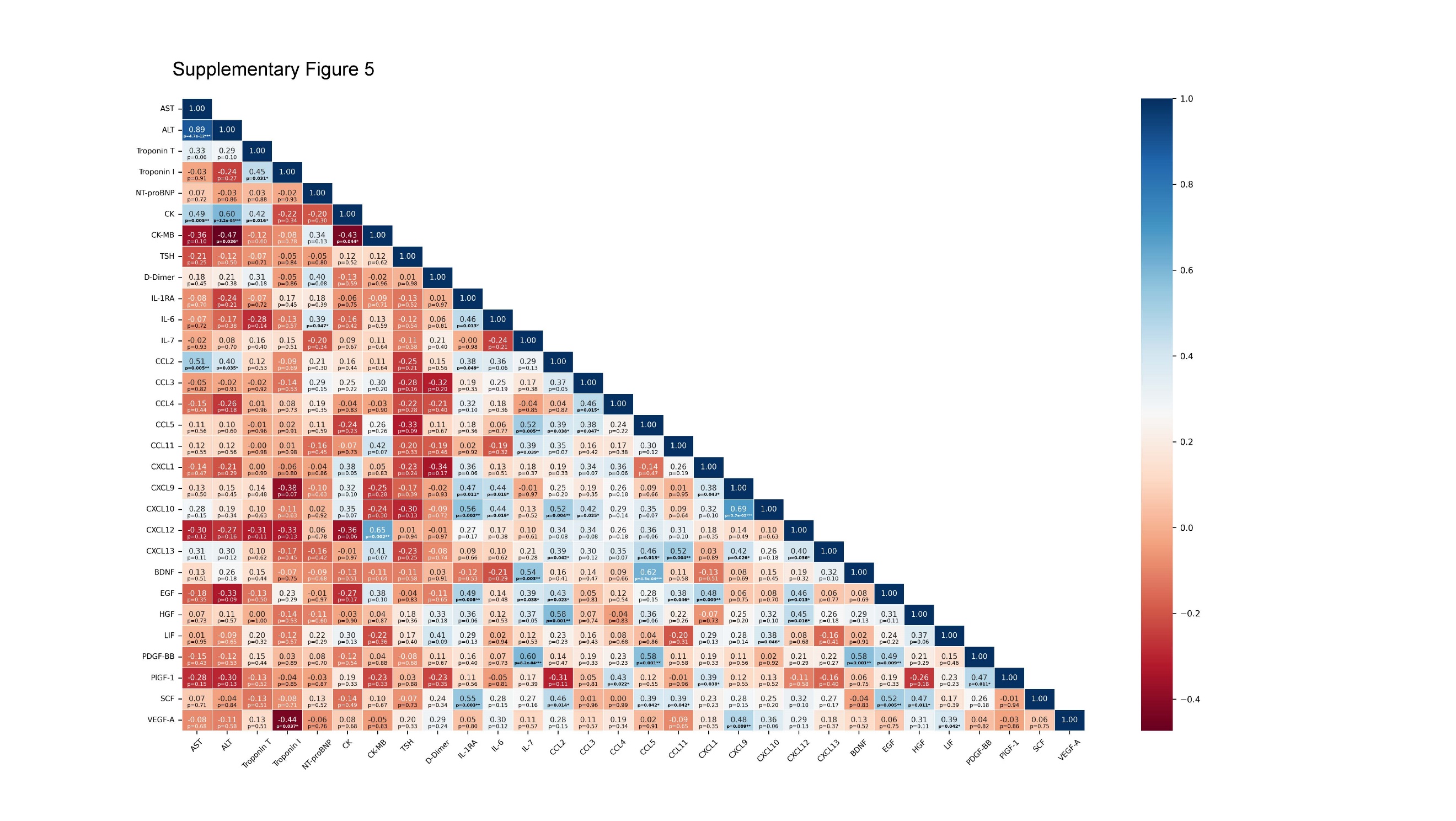
**

Supplemental Figure 5: Correlations between cytokines and conventional biological biomarkers in ICI-My. Spearman Correlation Matrix. Correlation map plotted using significance levels for the Spearman test performed with relevant serum biomarker data from all ICI-My patients studied across all grades. Positive correlations are shown in shaded blue and negative correlations are shown in shaded red. Correlations with a p-value ≥ 0.05 are not considered significant and are left blank. Color intensities are proportional to the correlation coefficients. On the right side of the correlogram, the color legend shows the correlation coefficients and corresponding colors


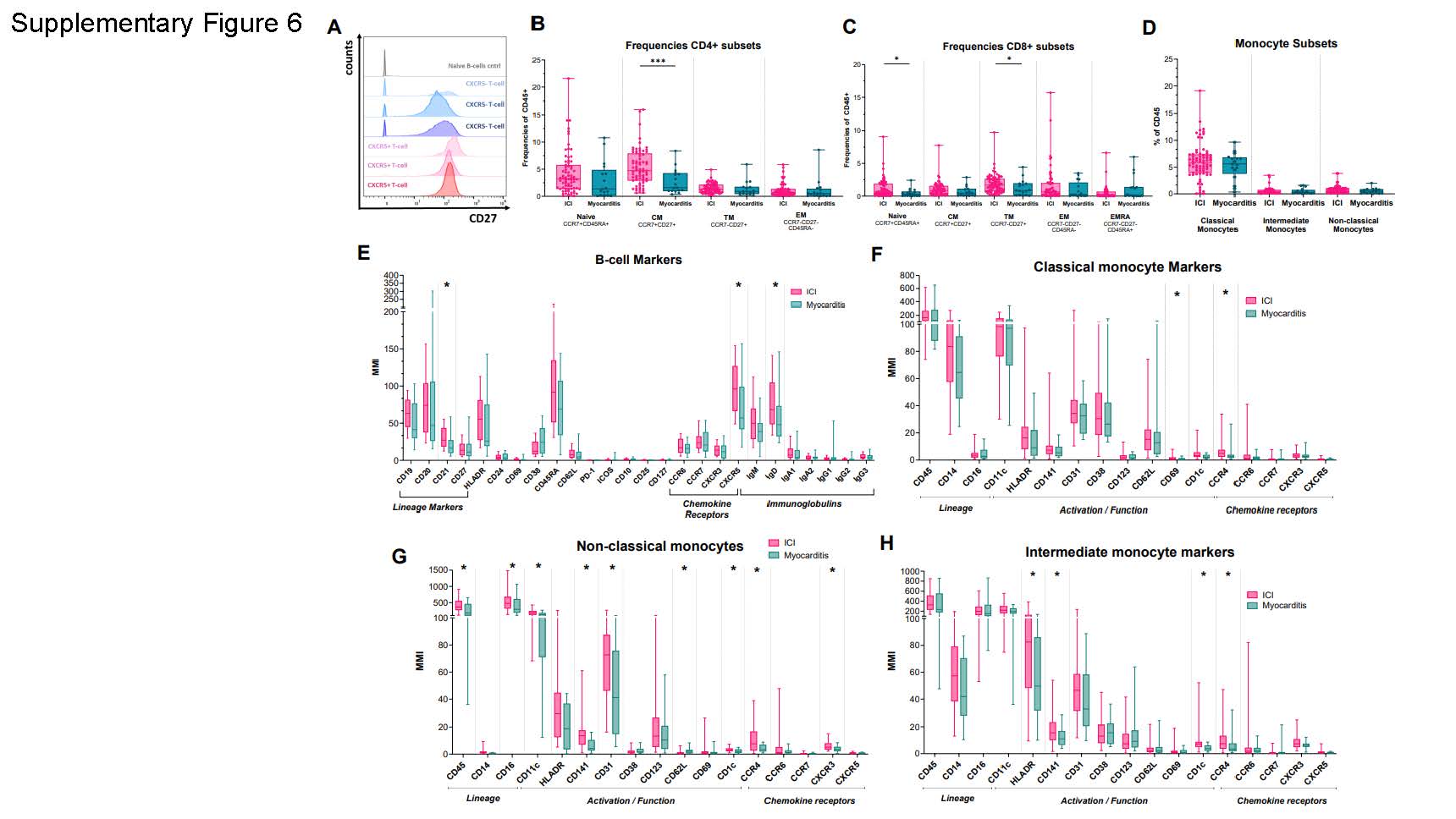


****Supplemental Figure 6.**** Distribution and phenotypes of circulating immune-cell subsets at myocarditis onset. (A) Frequencies of CXCR5+ B cells among total B cells (CD19+CD20+), naïve B cells (IgD+CD27-), and memory B cells (total minus naïve) in ICI-treated controls and patients with myocarditis. (B) Frequencies of CXCR5+ T cells among total T cells (CD3+), CD4+/CD8+ naïve T cells (CCR7+CD45RA+), and CD4+/CD8+ memory T cells. (C) Representative histograms showing CD27 expression in CXCR5+ (red) and CXCR5- (blue) T cells from 3 representative ICI-treated controls; the negative control is shown in gray (naïve B cells). (D-E) Frequencies of CD4+ and CD8+ T-cell subsets as a percentage of CD45+ cells. (F) Frequencies of monocyte subsets (classical CD14+CD16-, intermediate CD14+CD16+, and nonclassical CD14-CD16+) as a percentage of CD45+ cells. (G) Marker expression on total B cells (CD19+CD20+), expressed as mean metal intensity (MMI). (H-J) Marker expression on classical, intermediate, and nonclassical monocytes, expressed as MMI. Data were obtained from 16 patients at ICI-My diagnosis and compared with corresponding values from 72 ICI-treated cancer controls without myocarditis. Statistical significance was assessed using the Mann-Whitney U test, with *P<0.05 and **P<0.01.


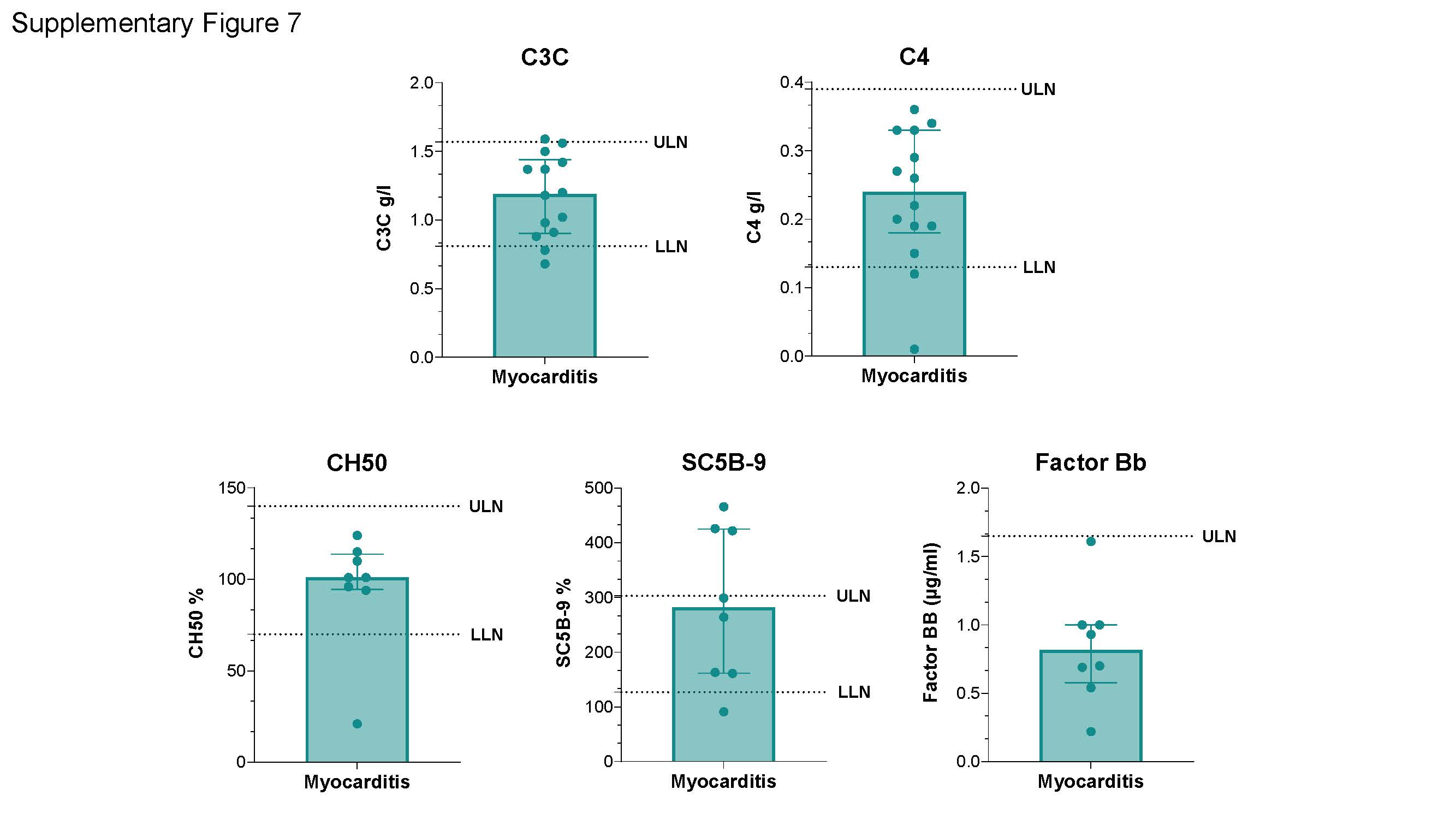


Supplemental Figure 7. Evaluation of complement-system involvement in ICI-My. Levels of key components representing the classical (C4, n=14; CH50, n=8), alternative (Factor Bb, n=8; C3c, n=14), and terminal (C5-b9, n=8) complement activation pathways were measured at ICI-My diagnosis.


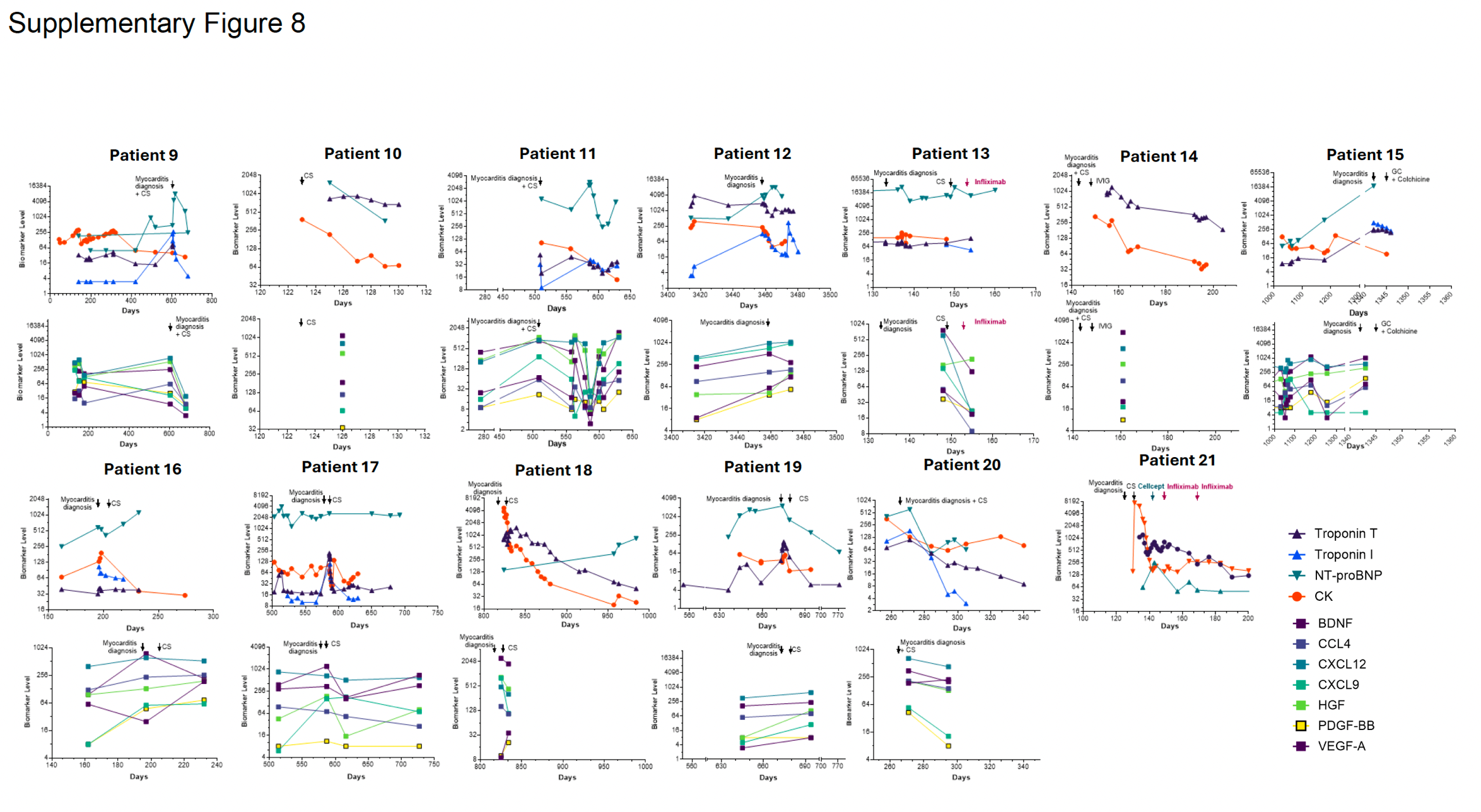


Supplemental Figure 8. Longitudinal biomarker changes and evidence of biological resolution in ICI-My treated with corticosteroids alone or in combination with other immunosuppressive agents, excluding tocilizumab. Each panel represents an individual patient (n=16) receiving either CS only or a combination of CS with other IS therapies, excluding TCZ. Longitudinal trajectories of key cardiac biomarkers (cTnT [ng/L], NT-proBNP [ng/L]) and corresponding changes in selected cytokines, chemokines, and growth factors are depicted over time. The date of myocarditis onset and the timing of immunosuppressive treatments are indicated by arrows on each patient’s panel, illustrating how these interventions influence biomarker profiles and potentially contribute to biological resolution. Abbreviations: CS, corticosteroids.


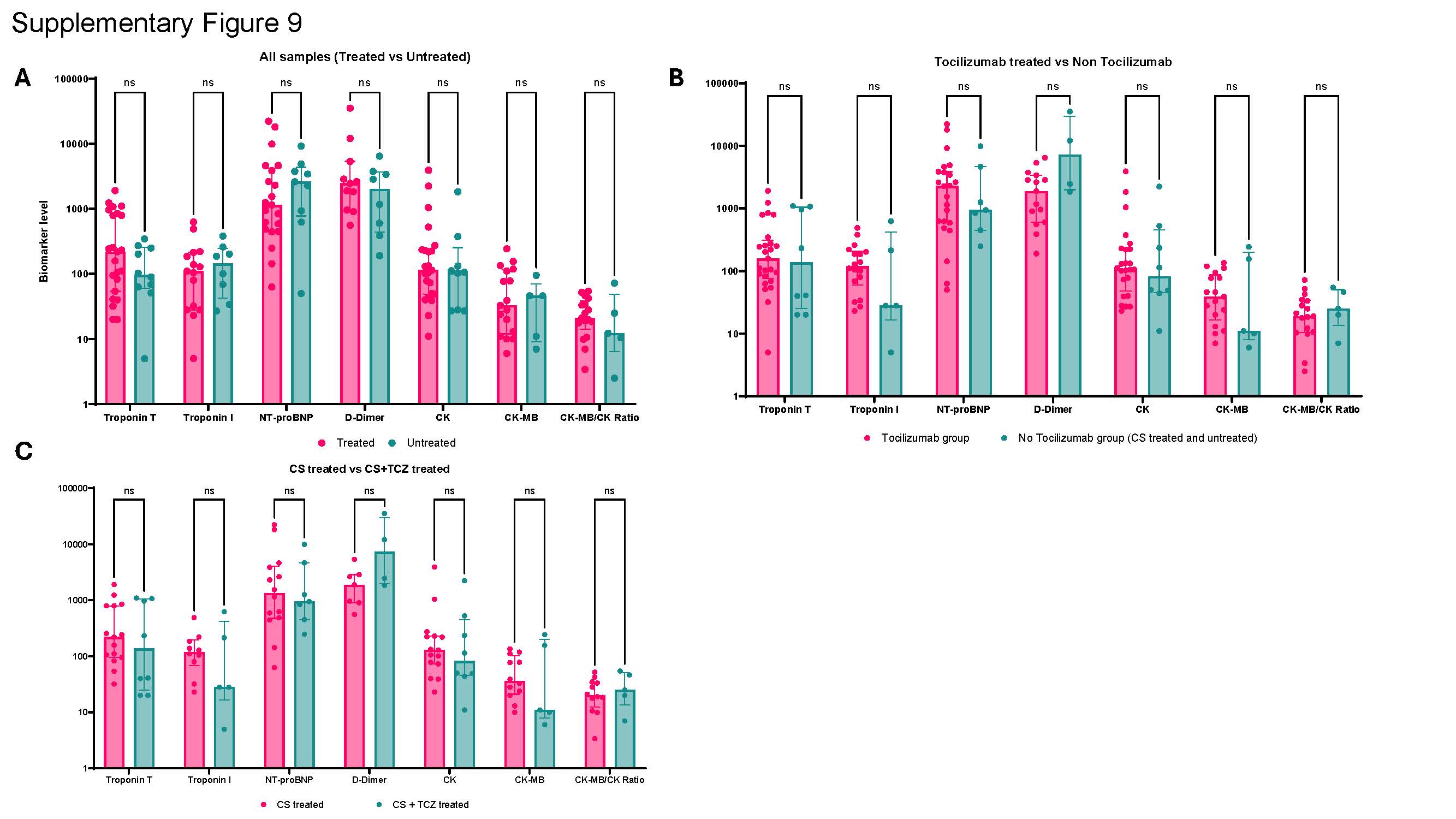


Supplemental Figure 9. Comparison of classical biomarkers in patients regarding their need for therapy. Classical biomarkers in ICI-My patients have been compared in 3 groups, (A) receiving any immunosuppressive treatment (n=23) or no immunosuppressive treatment (n=10), (B) all patients non receiving tocilizumab (n=25) or receiving tocilizumab (n=8) (C) receiving corticosteroids (n=15) or receiving corticosteroid plus tocilizumab (n=8). Comparison was performed on serum cardiac troponin T (cTnT) (A n=33, B n=33, C n=23), serum cardiac troponin I (cTnI) (A n=23, B n=23, C n=15), NT-proBNP (A n=30, B n=30, C n=21), D-dimers (A n=19, B n=19, C n=11), Total creatine kinase (CK) (A n=32, B n=32, C n=23), Creatine kinase-MB (CK-MB) (A n=22, B n=22, C n=17) and CK-MB/CK ratio (A n=22, B n=22, C n=17) in high and low grade ICI-My diagnosis. Bars represent the median and interquartile range (IQR). Each data point corresponds to an individual patient.

| **Characteristic** | **All unique ICI-treated controls (n=84)** | **Cytokine cohort (n=68)** | **CyTOF cohort (n=72)** |
| --- | --- | --- | --- |
| **Assay availability** |  |  |  |
| Unique control patients, n | 84 | 68 | 72 |
| Cytokines only | 12 (14.3) | 12 (17.6) | — |
| CyTOF only | 16 (19.0) | — | 16 (22.2) |
| Both cytokines and CyTOF | 56 (66.7) | 56 (82.4) | 56 (77.8) |
| **Age** |  |  |  |
| Age, years | 65 [56–75] | 66 [57–76] | 65 [59–75] |
| Age ≥65 years, n (%) | 44 (52.4) | 37 (54.4) | 39 (54.2) |
| **Sex** |  |  |  |
| Female sex, n (%) | 41 (48.8) | 31 (45.6) | 33 (45.8) |
| Male sex, n (%) | 43 (51.2) | 37 (54.4) | 39 (54.2) |
| **Tumor type** |  |  |  |
| Lung cancer | 38 (45.2) | 30 (44.1) | 35 (48.6) |
| Melanoma | 23 (27.4) | 19 (27.9) | 19 (26.4) |
| Breast cancer | 13 (15.5) | 11 (16.2) | 9 (12.5) |
| Other tumor types | 10 (11.9) | 8 (11.8) | 9 (12.5) |
| **ICI regimen** |  |  |  |
| Dual-checkpoint blockade | 19 (22.6) | 18 (26.5) | 15 (20.8) |
| Ipilimumab + nivolumab | 19 (22.6) | 18 (26.5) | 15 (20.8) |
| Single-agent ICI | 65 (77.4) | 50 (73.5) | 57 (79.2) |
| Pembrolizumab | 46 (54.8) | 35 (51.5) | 39 (54.2) |
| Atezolizumab | 6 (7.1) | 5 (7.4) | 6 (8.3) |
| Durvalumab | 6 (7.1) | 3 (4.4) | 6 (8.3) |
| Nivolumab | 5 (6.0) | 5 (7.4) | 4 (5.6) |
| Cemiplimab | 1 (1.2) | 1 (1.5) | 1 (1.4) |
| Retifanlimab | 1 (1.2) | 1 (1.5) | 1 (1.4) |
| **Stage at diagnosis** |  |  |  |
| Stage I–III | 49 (58.3) | 38 (55.9) | 43 (59.7) |
| Stage IV | 33 (39.3) | 28 (41.2) | 27 (37.5) |
| Not staged | 2 (2.4) | 2 (2.9) | 2 (2.8) |
| **Sampling timing** |  |  |  |
| Time from first ICI dose to sampling, days | — | 29 [28–42] | 28 [27–42] |

Supplementary Table 1: ICI-treated control cohort. A total of 84 unique ICI-treated control patients without myocarditis or other clinically apparent irAEs were included. Of these, 68 had cytokine data, 72 had CyTOF data, and 56 had both assays available. Sampling was performed predominantly around cycle 2 of treatment. Therefore, the cytokine and CyTOF subcohorts partially overlapped and were not mutually exclusive. Sampling was performed predominantly around cycle 2 of treatment.

| **Supplementary Table 2** |  | **N=8** | **% within cohort** |
| --- | --- | --- | --- |
| **Tumor type** |  |  |  |
| Melanoma |  | 3 | 37% |
| Lung |  | 2 | 24% |
| Breast |  | 1 | 13% |
| Kidney |  | 1 | 13% |
| Prostate |  | 1 | 13% |
| **ICI treatment** |  |  |  |
|  |  |  |  |
| Anti-CTLA4/PD1 |  | 6 | 75% |
| Anti-PD1 |  | 2 | 25% |
| **Associated irAEs** |  |  |  |
| Colitis |  | 4 | 50% |
| Myocarditis-myositis overlap syndrome |  | 1 | 13% |
| CRS |  | 1 | 13% |
| Cholangio-hepatitis |  | 1 | 13% |
| **Myocarditis grading at diagnosis** |  |  |  |
| Non-severe |  | 5 | 63% |
| Severe |  | 3 | 38% |
| **Previous immunosupression therapy** |  |  |  |
| High-dose steroids (HDS) |  | 8 | 100% |
| Infliximiab (IFX) |  | 2 | 25% |
| Mycophenolic mofetil (MMF) |  | 1 | 13% |
| **Tocilizumab therapy** |  |  |  |
|  |  |  |  |
| Mortality related to myocarditis |  | 0 | 0% |
| Clinical improvement |  | 8 | 100% |
| Biological and cytokine improvement (n=7) |  | 7 | 100% |
| CS tapering |  | 8 | 100% |
| 1 Dose of tocilizumab administered |  | 4 | 50% |
| 2 Dose of tocilizumab administered |  | 1 | 13% |
| 3 Dose of tocilizumab administered |  | 3 | 38% |
| **Biomarkers Values** |  | **mean (SD)** | **n** |
| CK (U/l) |  |  |  |
| Maximum before TCZ |  | 695.9 (1251) | 7 |
| Minimum after TCZ |  | 43 (23.21) | 7 |
| Fold change (SD) |  | 14.43 (18.67) | 6 |
| CK-MB (U/l) |  |  |  |
| Maximum before TCZ |  | 87.4 (106.9) | 5 |
| Minimum after TCZ |  | 31.5 (5.447) | 4 |
| Fold change (SD) |  | 4.023 (3.383) | 3 |
| NT-proBNP (ng/l) |  |  |  |
| Maximum before TCZ |  | 2611 (3528) | 7 |
| Minimum after TCZ |  | 712.2 (615.8) | 6 |
| Fold change (SD) |  | 10.67 (17.8) | 6 |
| Troponine I (ng/l) |  |  |  |
| Maximum before TCZ |  | 424.6 (505.4) | 5 |
| Minimum after TCZ |  | 81 (149.4) | 4 |
| Fold change (SD) |  | 19.6 (18.69) | 4 |
| Troponine T (ng/l) |  |  |  |
| Maximum before TCZ |  | 581.1 (731.4) | 8 |
| Minimum after TCZ |  | 100.3 (134.7) | 7 |
| Fold change (SD) |  | 8.552 (7.331) | 7 |

Supplementary Table 2: Clinical characteristics of ICI-My patients receiving tocilizumab treatment (n=8).

Supplementary Table 3: Table summarizing descriptive statistics (N, mean, 25th percentile, median, 75th percentile, and SD) and lower limits of detection for all cytokines, chemokines, and growth factors, stratified by myocarditis severity when applicable. (Full size excel file uploaded separately)

Supplementary Table 4. Patient-level characteristics of the ICI-treated control cohort by assay availability. A total of 84 unique ICI-treated control patients without myocarditis were included. (Full size excel file uploaded separately)

Supplemental References

1. Noto A, Suffiotti M, Joo V et al. The deficiency in Th2-like Tfh cells affects the maturation and quality of HIV-specific B cell response in viremic infection. Front Immunol 2022;13:960120.

2. Daoudlarian D SA, Latifyan S, Bartolini R, Joo V, Mederos N, Bouchaab H, Demicheli R, Abdelhamid K, Ferahta N, Doms J, Stalder G, Noto A, Mencarelli L, Mosimann V, Berthold B, Stravodimou A, Sartori C, Shabafrouz K, Thompson JA, Wang Y, Peters S, Pantaleo G, Obeid M. Tocilizumab and immune signatures for targeted management of cytokine release syndrome in immune checkpoint therapy. Annals of Oncology 2024;In press.

3. Joo V, Abdelhamid K, Noto A et al. Primary prophylaxis with mTOR inhibitor enhances T cell effector function and prevents heart transplant rejection during talimogene laherparepvec therapy of squamous cell carcinoma. Nat Commun 2024;15:3664.

4. Ozdemir BC, Latifyan S, Perreau M et al. Cytokine-directed therapy with tocilizumab for immune checkpoint inhibitor-related hemophagocytic lymphohistiocytosis. Ann Oncol 2020;31:1775-1778.

5. Doms J, Prior JO, Peters S, Obeid M. Tocilizumab for refractory severe immune checkpoint inhibitor-associated myocarditis. Ann Oncol 2020;31:1273-1275.

6. Moi L, Bouchaab H, Mederos N et al. Personalized Cytokine-Directed Therapy With Tocilizumab for Refractory Immune Checkpoint Inhibitor-Related Cholangiohepatitis. J Thorac Oncol 2021;16:318-326.

7. Petit PF, Daoudlarian D, Latifyan S et al. Tocilizumab provides dual benefits in treating immune checkpoint inhibitor-associated arthritis and preventing relapse during ICI rechallenge: the TAPIR study. Ann Oncol 2024.
